# SphK1/mitophagy axis in cementocytes drives orthodontic root resorption via mitochondrial transfer to osteoclasts

**DOI:** 10.1038/s41413-026-00538-0

**Published:** 2026-05-14

**Authors:** Han Wang, Sihang Chen, Shuo Chen, Chengchen Duan, Li Zhu, Hengyi Lin, Shujuan Zou, Yu Li, Peipei Duan

**Affiliations:** 1 State Key Laboratory of Oral Diseases & National Center for Stomatology & National Clinical Research Center for Oral Diseases & Department of Orthodontics, West China Hospital of Stomatology, Sichuan University, Chengdu, Sichuan China; 2 Clinical Research Center for Oral Tissue Deficiency Diseases of Fujian Province & Fujian Key Laboratory of Oral Diseases & Fujian Provincial Engineering Research Center of Oral Biomaterial, School and Hospital of Stomatology, Fujian Medical University, Fuzhou, China; 3 Stomatological Key laboratory of Fujian College and University & Institute of Stomatology & Department of Orthodontics, School and Hospital of Stomatology, Fujian Medical University, Fuzhou, China

**Keywords:** Bone quality and biomechanics, Dental diseases

## Abstract

Orthodontically induced inflammatory root resorption (OIIRR) is a prevalent complication driven by excessive mechanical force, yet the underlying mechanisms linking mechanotransduction to osteoclast activation remain elusive. Here, we identify a novel signaling axis wherein sphingosine kinase 1 (SphK1) in cementocytes translates heavy orthodontic force into a pro-osteoclastogenic signal via mitophagy-mediated mitochondrial transfer. In vivo, heavy force induced OIIRR and upregulated mitophagy markers in cementocytes. In vitro, heavy compression force triggered SphK1-dependent mitophagy in IDG-CM6 cementocytes, as evidenced by increased mitophagosome formation, co-localization of mitochondria with lysosomes, and elevated PINK1/PARKIN signaling. Inhibition of SphK1, either pharmacologically or genetically, suppressed this mitophagic response. Conditioned media from force-loaded cementocytes enhanced osteoclast differentiation and glycolytic metabolism, effects that were abolished by SphK1 inhibition and rescued by a mitophagy agonist. Crucially, we demonstrated that heavy force promotes the transfer of mitochondria from cementocytes to osteoclast precursors, a process dependent on mitophagy. This transferred mitochondrial cargo functioned as a metabolic subsidy, boosting osteoclast bioenergetics and resorptive activity. Our findings unveil the SphK1-mitophagy-mitochondrial transfer axis as a fundamental mechanism of cementocyte-osteoclast communication, positioning SphK1 as a promising therapeutic target to prevent OIIRR.

## Introduction

Orthodontic treatment has become increasingly prevalent worldwide due to rising demand.^1^ However, orthodontically induced inflammatory root resorption (OIIRR) remains a common and concerning complication, with a relatively high incidence (48%–66% for cases involving 2 mm or less, and 1%–5% for cases with more than 4 mm of root resorption).^2,3^ OIIRR shortens the longevity of teeth, thereby impairing masticatory function and potentially leading to serious health issues, including malnutrition.^4^ Furthermore, it can adversely affect the doctor-patient relationship.^5^ The challenge for orthodontists lies in the variability of optimal force requirements across different teeth and individuals, making it difficult to consistently apply the precise force needed.^6^ As a result, excessive orthodontic force, a known trigger for OIIRR,^6,7^ may be inadvertently applied during treatment.^6^ Therefore, there is an urgent need to elucidate the molecular mechanisms underlying OIIRR and to identify effective therapeutic targets to mitigate this condition.

Osteoclasts, arisen by differentiation of monocyte/macrophage lineage precursors, are the primary effector cells responsible for initiating the pathological process of OIIRR.^8,9^ Growing evidence suggests that cell-to-cell communication plays a crucial role in the activation of osteoclasts during their regulation on OIIRR.^7,10–12^ Cementum, a mineralized connective tissue covering the root surface, is the initial tissue and primary site to undergo resorption in OIIRR.^13^ Unlike cementoblasts which are actively involved in the repair of root resorption,^14^ cementocytes, the primary mechanically responsive cells within cementum, have been implicated in the regulation of osteoclastogenesis through intercellular communication.^7,15,16^ Despite the documentation of complex interactions between these cellular lineages, the underlying regulatory mechanisms remain incompletely understood.

Mitochondria, often referred to as the energy factories of eukaryotic cells, have emerged as critical contributors to cellular metabolism and differentiation, particularly in bone biology.^17,18^ Mitophagy, a key cellular process, maintains mitochondrial homeostasis by eliminating superfluous or damaged mitochondria that could impede cell differentiation and survival.^19,20^ Highly metabolic tissues, such as bone, maintain steady-state levels of mitophagy, but this process is also activated in response to internal or external stress stimuli.^21,22^ Intriguingly, a recent study has demonstrated that certain cells undergoing mitophagy in response to oxidative stress can package mitochondria for intercellular transfer, thereby enhancing the bioenergetics and functions of recipient cells.^23^ Intercellularly transferred mitochondria, functioning as signaling organelles, have increasingly been recognized as a novel mechanism of information exchange between cells.^24^ Several studies have reported that osteocytes, through their extensive network of dendritic processes, can donate mitochondria to neighboring cells, thereby synchronizing energy metabolism.^25–27^ Given that cementocytes possess an osteocyte-like lacuno-canalicular system,^28^ we hypothesize that cementocytes may regulate the energy metabolism and biological activity of osteoclasts via mitochondrial transfer.

Sphingosine-1-phosphate (S1P) is a bioactive lysophospholipid that regulates various physiological processes and mediates signal transduction in mammalian cells.^13^ Sphingosine kinase (SphK) is critical for S1P biosynthesis, with SphK1 being the predominant isoform involved in bone metabolism.^29,30^ As a key mediator of osteoclastogenesis, the SphK1/S1P axis has been shown by our group and others to promote osteoclast differentiation and mineralized tissue resorption through intercellular communication.^7,31^ SphK1 expression in osteocytes is known to be essential for mitochondrial transfer to recipient cells.^27^ However, whether this SphK1-mediated organelle transfer occurs in cementocyte-osteoclast communication remains to be fully elucidated.

In this study, we identified a previously unrecognized role of SphK1 in cementocyte-osteoclast communication. We observed that force-loaded cementocytes underwent SphK1-mediated mitophagy, resulting in the release of mitochondria, which were subsequently engulfed by osteoclast precursors (OCPs). This mitochondrial transfer enhanced the biological activity and glycolytic metabolism of osteoclasts. Inhibition of SphK1 in force-loaded cementocytes suppressed mitophagy, thereby reducing mitochondrial transfer to osteoclasts and mitigating OIIRR. Collectively, our findings suggest that SphK1-mediated mitophagy in force-loaded cementocytes facilitates mitochondrial transfer to osteoclasts, representing a previously unappreciated mechanism that orchestrates osteoclastogenesis and provides new insights into the progression of OIIRR.

## Results

### Heavy orthodontic force induces OIIRR and is associated with enhanced mitophagy in cementocytes

**Fig. 1:**
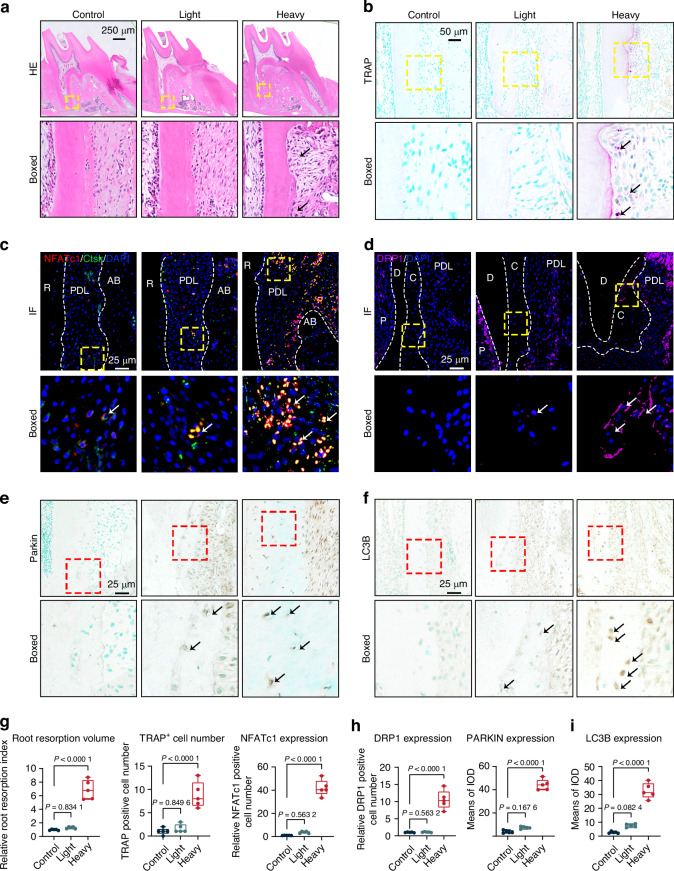
Heavy orthodontic force induces OIIRR and is associated with enhanced mitophagy in cementocytes. **a** Representative HE staining images of the distobuccal roots of M1 under 100 g orthodontic force at Day 14. The black arrow indicated the resorption pits. Scale bar = 250 μm. **b** Osteoclasts at the mesial surface of distobuccal roots were stained with TRAP. The black arrow indicated the osteoclasts at the interested area. R, root. Scale bar = 50 μm. **c** Representative immunofluorescence images of NFATc1 (red) at the mesial surface of distobuccal roots. The Ctsk (green) was used to label osteoclasts. The yellow box indicated the interested area at the lower panel. The white arrow indicated the osteoclasts at the interested area. R, Root; PDL, Periodontal ligament; AB, Alveolar bone. Scale bar = 25 μm. **d** Representative immunofluorescence images of DRP1 (magenta) in cementocytes at the mesial surface of distobuccal roots. The yellow box indicated the interested area at the lower panel. The white arrow indicated the cementocytes at the interested area. Scale bar = 25 μm. **e** Representative immunohistochemical images of PARKIN at the mesial surface of distobuccal roots. The red box indicated the interested area at the lower panel. The black arrow indicated the positive cells at the interested area. Scale bar = 25 μm. **f** Representative immunohistochemical images of LC3B at the mesial surface of distobuccal roots. The red box indicated the interested area at the lower panel. The black arrow indicated the positive cells at the interested area. Scale bar = 25 μm. **g**–**i** The M1 distobuccal root resorption volume, quantification of TRAP-positive cells number per millimeter root length and quantitative analysis of NFATc1-, DRP1-positive cells, as well as PARKIN and LC3B positive staining, at the interested area. Statistical comparison was performed using one-way ANOVA with Tukey’s post hoc test. *P* < 0.05 was considered statistically significant. *n* = 5 for each group. All data were presented as mean ± SEM

To investigate the effects of heavy orthodontic force on root cementum during orthodontic tooth movement (OTM) in vivo, we first analyzed the morphology of the root surface. Compared with the Control and Light orthodontic force groups, a greater number of resorption pits and irregularly compressed periodontal fibers were observed on the compression side of distobuccal roots subjected to heavy orthodontic force, as demonstrated by HE and Masson’s trichrome staining (Figs. 1a and S1a). Consistently, the volume of resorption lacunae and the number of TRAP-positive cells were significantly increased in the Heavy orthodontic force group, along with stronger expression of NFATc1 in osteoclasts labeled with Ctsk (Figs. 1b, c, g and S1b).^7^ Given that mitophagy has been shown by us and others to regulate periodontal homeostasis and mineralized tissue resorption,^22,32^ we next examined the mitophagy-associated markers, DRP1 and PARKIN, in cementocytes.^19^ We observed a greater number of DRP1-positive cementocytes and PARKIN-positive staining in the Heavy group compared to the Control and Light groups (Fig. 1d, e, h). Additionally, the expression of LC3B, a specific marker of autophagy and mitophagy,^19,33^ was also elevated in cementocytes under heavy orthodontic force relative to the other two groups (Fig. 1f, i). Collectively, these findings indicate that heavy orthodontic force induces OIIRR, which is associated with enhanced mitophagy in cementocytes, suggesting a close association between mitophagy in cementocytes and heavy force-induced OIIRR.

### Heavy compression force enhances mitophagy in IDG-CM6 cells

**Fig. 2:**
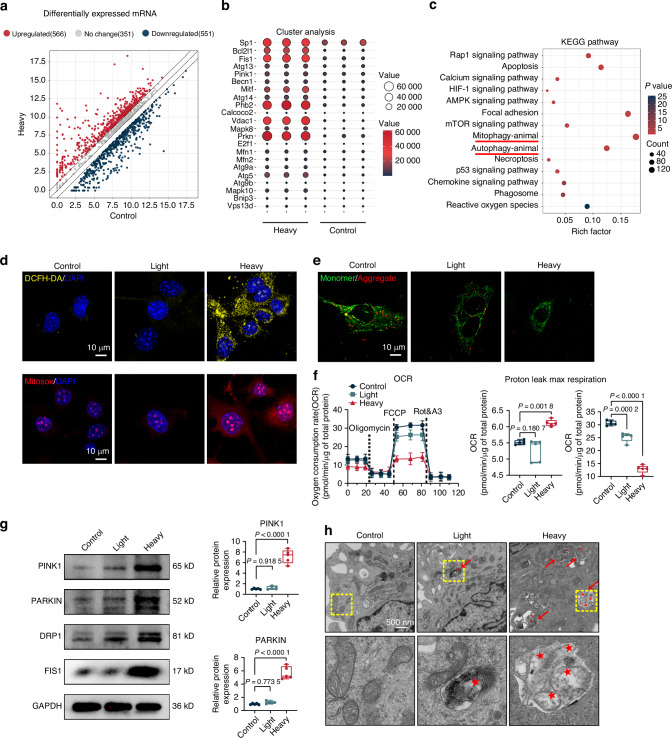
Heavy compression force enhances mitophagy in IDG-CM6 cells. **a** The scatter plot displayed global gene expression in the IDG-CM6 cells under heavy compression force. **b** The heatmap summarized the significantly expressed mitophagy-related genes. **c** Kyoto Encylopaedia of Genes and Genomes (KEGG) analysis showing significantly enriched mitophagy- and autophagy-related signaling pathways. **d** Representative images of cellular ROS production (yellow fluorescence) and MitoSOX-stained mitochondrial ROS (red fluorescence) in IDG-CM6 cells after heavy compression force loading. Scale bar = 10 μm. **e** Representative images of JC-1 assay in IDG-CM6 cells after heavy compression force loading. Scale bar = 10 μm. **f** The oxygen consumption rate (OCR) was recorded. The OCR was normalized to the relative cell counts. Representative OCR time-course data and associated data based on the OCR were shown. **g** The protein expression of PINK1, PARKIN, DRP1 and FIS1 were examined by western blot analysis using GAPDH as a loading control. The quantitative analysis of protein expression of PINK1 and PARKIN was also performed. **h** Transmission electron microscopy (TEM) images and quantitative analysis of mitophagosomes in IDG-CM6 cells. The yellow box indicated the interested area at the lower panel. The red arrow indicates the mitophagosome. The asterisk indicated the mitochondria incorporated into autolysosomes. Scale bars = 500 nm. Statistical comparison was performed using one-way ANOVA with Tukey’s post hoc test. *P* < 0.05 was considered statistically significant. *n* = 5 for each group. All data were presented as mean ± SEM

To evaluate whether heavy compression force stimulates mitophagy in cementocytes in vitro, we conducted RNA sequencing (RNA-seq) on IDG-CM6 cells subjected to heavy compression force for 6 h. The scatter plots and heatmap analysis revealed distinct differences in gene expression patterns between the Control and Heavy groups (Fig. 2a). Further cluster analysis identified significantly upregulated mitophagy-related genes, including *Pink1*, *Prkn*, *Fis1*, and *Phb2*, in the Heavy group (Figs. 2b and S2a). KEGG pathway analysis indicated a significant enrichment of mitophagy- and autophagy-related signaling pathways, with mitophagy being the most prominently enriched (Fig. 2c). Consistently, Gene Ontology (GO) analysis, encompassing Biological Process (BP), Cellular Component (CC), and Molecular Function (MF), showed that differentially expressed mRNAs were enriched in processes related to mitochondrial bioenergetics and dynamics (Fig. S2b). Given the well-established role of mitochondrial dysfunction in the indication of mitophagy,^19,34^ we assessed intracellular reactive oxygen species (ROS), mitochondrial ROS (mtROS), and mitochondrial membrane potential (MMP) in IDG-CM6 cells. Heavy force triggered significant oxidative stress, marked by increased ROS and mtROS, and led to mitochondrial dysfunction as evidenced by a reduced MMP and decreased OCR across parameters of basal respiration, ATP production, and respiratory capacity (Figs. 2d–f and S2c). A slight increase in proton leak was also observed (Fig. 2f). In line with these findings, the expression levels of key autophagy-related proteins including ATG5, ATG7, Beclin1, and LC3B, which serve as a prerequisite for executing mitophagy,^19^ were significantly elevated (Fig. S3). Additionally, the mRNA and protein levels of key mitophagy markers, PINK1 and PARKIN, as well as DRP1 and FIS1,^19^ were significantly upregulated in the Heavy group, indicating enhanced mitophagy in cementocytes under heavy force (Figs. 2g and S4a, c). To further substantiate these observations, transmission electron microscopy (TEM) revealed a substantial increase in mitophagosomes in IDG-CM6 cells exposed to heavy force compared to controls and light force conditions (Fig. 2h). Consistent with this, immunofluorescence co-localization of the lysosomal marker LAMP1^19^ and mitochondria labeled with TOMM20^35^ demonstrated a higher degree of mitochondria-lysosome co-localization in heavy force-loaded cells compared to controls and light force (Fig. S4b). This enhanced lysosomal engagement was further evidenced by a similar increase in the co-localization of LAMP1 with the mitophagy regulators PINK1, PARKIN, DRP1, and FIS1 (Fig. S4d). Collectively, these findings suggest that heavy compression force promotes mitophagy in cementocytes.

### SphK1 promotes mitophagy in IDG-CM6 cells under heavy compression force

**Fig. 3:**
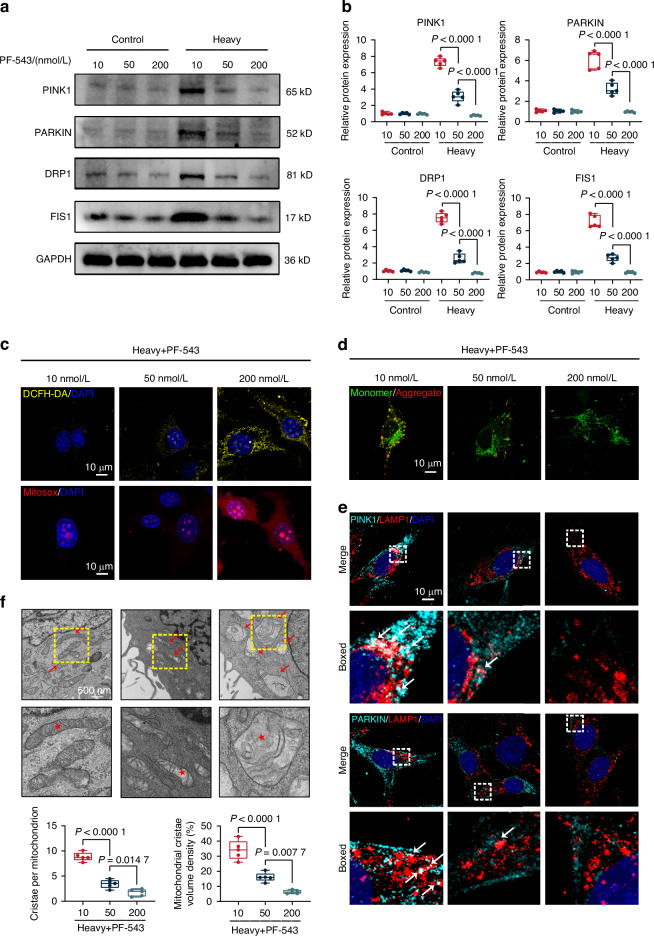
SphK1 promotes mitophagy in IDG-CM6 cells under heavy compression force. **a**, **b** The protein expression of PINK1, PARKIN, DRP1 and FIS1 were examined by western blot analysis using GAPDH as a loading control. The quantitative analysis of protein expression of PINK1, PARKIN, DRP1 and FIS1 was also performed. **c** Representative images of cellular ROS production (yellow fluorescence) and MitoSOX-stained mitochondrial ROS (red fluorescence) in IDG-CM6 cells after heavy compression force loading. Scale bar = 10 μm. **d** Representative images of JC-1 assay in IDG-CM6 cells after heavy compression force loading. Scale bar = 10 μm. **e** Representative coimmunostaining images were presented for PINK1, PARKIN, with LAMP1. The white box indicated the interested area at the lower panel. The white arrow indicated the colocalization of PINK1, PARKIN, DRP1 and FIS1 with lysosomes, respectively. Scale bar = 10 μm. **f** TEM images and quantitative analysis of swelling mitochondrial and cristae disruption in IDG-CM6 cells. The yellow box indicated the interested area at the lower panel. The red arrow and the asterisk indicate the swelling and cristae disrupted mitochondria. Scale bars = 500 nm. Statistical comparison was performed using two-tailed Student’s *t* test or one-way ANOVA with Tukey’s post hoc test. *P* < 0.05 was considered statistically significant. *n* = 5 for each group. All data were presented as mean ± SEM

Our preliminary study identified SphK1 as a critical mechanosensor and mechanotransducer in cementocytes subjected to heavy force loading, corroborating previous findings on its role in the regulation of mitophagy induced by external stimuli.^7,36–38^ Consequently, we investigated whether SphK1 mediates mitophagy in IDG-CM6 cells under heavy compressive force. We first observed that treatment with the SphK1-specific inhibitor PF-543^39^ suppressed the expression of PINK1, PARKIN, DRP1, and FIS1 in a dose-dependent manner in heavy force-loaded IDG-CM6 cells (Figs. 3a, b and S5a). This finding suggests that SphK1 is essential for heavy force-induced mitophagy in cementocytes. To further solidate this mechanism, we measured ROS, mtROS, and MMP levels, and conducted immunofluorescence co-localization studies. Our results demonstrated that, under heavy compression force, PF-543 induced the generation of intracellular ROS and mtROS, reduced JC-1 staining, and decreased the co-localization of PINK1, PARKIN, DRP1, and FIS1 with LAMP1, all in a dose-dependent manner (Figs. 3c–e and S5b). Consistent with these findings, TEM analysis revealed that PF-543 significantly increased mitochondrial swelling and cristae disruption under heavy compressive force (Fig. 3f), further supporting a modulatory role of SphK1 in heavy force-induced mitophagy.

To directly assess the involvement of SphK1 in force-induced mitophagy, we employed small interfering RNA (siRNA) to knock down SphK1 expression in IDG-CM6 cells. The pronounced upregulation of PINK1, PARKIN, DRP1, and FIS1 induced by heavy compression force was partially reversed by SphK1 suppression (Figs. 3g, h and S5c, d). Consistently, immunofluorescence co-localization analysis showed that SphK1 suppression significantly reduced the co-localization of PINK1, PARKIN, DRP1, and FIS1 with LAMP1 under heavy compressive force (Fig. S6). Collectively, these results confirm that SphK1 facilitates heavy force-induced mitophagy in cementocytes.

### SphK1-mediated mitophagy in heavy force-loaded IDG-CM6 cells regulates osteoclastogenesis and glycolytic metabolism

**Fig. 4:**
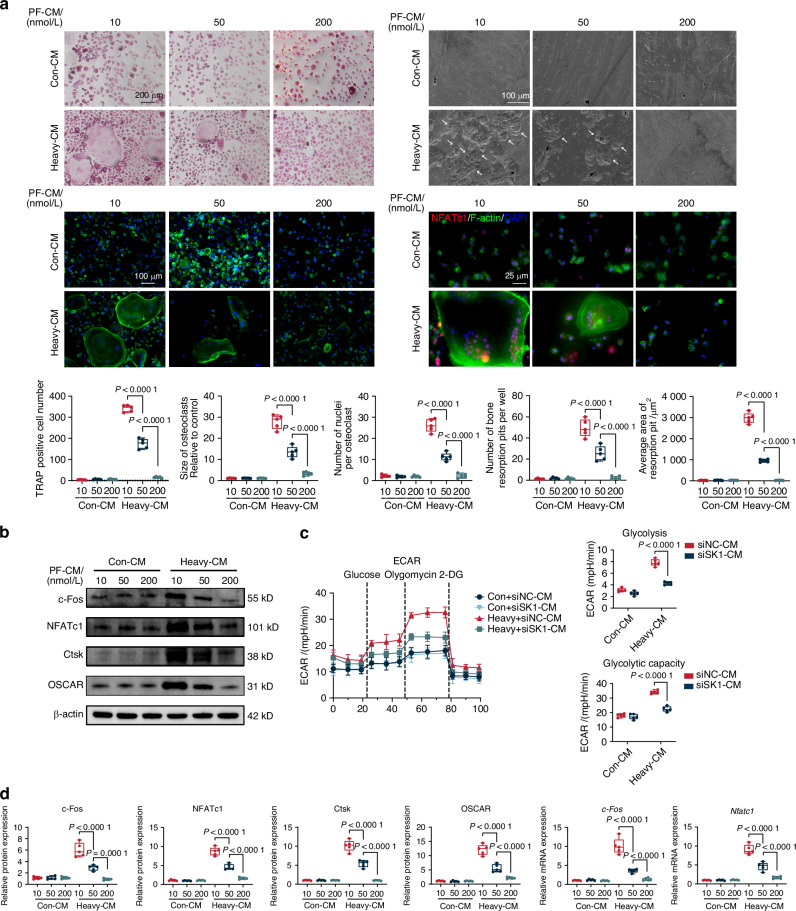
SphK1-mediated mitophagy in heavy force-loaded IDG-CM6 cells regulates osteoclastogenesis and glycolytic metabolism. **a** Representative images of TRAP staining. The arrows indicated osteoclasts. Scale bar = 200 μm. Representative images of F-actin ring staining. The arrows indicated osteoclasts. Scale bar = 100 μm. Representative immunofluorescence images of NFATc1 level in osteoclasts. Cytoskeleton, green; NFATc1, red; Nuclei, blue. Scale bar = 25 μm. The SEM of resorption pits on bovine slices seeded with differentiating osteoclasts were also performed, the white arrows indicated the resorption lacunae on bovine slices. Scale bar = 100 μm. Quantitative analysis of number of nuclei per osteoclast and quantification of number and relative size of TRAP positive multinucleated osteoclasts per well, as well as the quantification of number and average area of resorption pits, were performed and values were normalized to those in the Con-CM with 10 nmol/L PF-543 group. The PF-CM indicated the conditioned media collected from the heavy force-loaded IDG-CM6 cells treated with PF-543. **b** The protein expression of c-Fos, NFATc1, Ctsk and OSCAR in BMMs were examined by western blot analysis using GAPDH as a loading control. **c** The extracellular acidification rate (ECAR) was recorded. The ECAR was normalized to the relative cell counts. Representative ECAR time-course data and assessments of glycolysis and the glycolytic capacity based on the ECAR were shown. **d** The quantitative analysis of protein expression of c-Fos, NFATc1, Ctsk and OSCAR was performed using GAPDH as a loading control. Quantitative RT-PCR analysis was performed to examine the mRNA levels of *c-Fos* and *Nfatc1*. Statistical comparison was performed using two-tailed Student’s *t* test or one-way ANOVA with Tukey’s post hoc test. *P* < 0.05 was considered statistically significant. *n* = 5 for each group. All data were presented as mean ± SEM

Given the in vivo data suggesting that mitophagy is closely associated with heavy force-induced OIIRR initiated by osteoclasts,^8^ we further investigated whether SphK1-mediated mitophagy in heavy force-loaded IDG-CM6 cells regulates osteoclastogenesis. As previously described,^7^ conditioned media (CM) from heavy force-loaded IDG-CM6 cells were used as culture media for co-culturing with bone marrow-derived macrophages (BMMs). As shown in Fig. 4a, inhibition of SphK1 with PF-543 in heavy force-loaded IDG-CM6 cells significantly reduced osteoclastogenesis, resorption pits and the nuclear translocation of osteoclast-specific marker NFATc1,^7^ in a dose-dependent manner. Consistently, the expression of NFATc1 and other osteoclast-specific markers, including c-Fos, Ctsk, and OSCAR, was decreased in a dose-dependent manner when BMMs were cultured with CM from heavy force-loaded IDG-CM6 cells that had been treated with increasing concentrations of PF-543 (Figs. 4b, d and S7). Similarly, knockdown of SphK1 in IDG-CM6 cells produced effects on osteoclastogenesis that paralleled those observed with PF-543 treatment (Fig. S8). In summary, suppression of SphK1 in heavy force-loaded IDG-CM6 cells inhibits osteoclastogenesis and osteoclastic resorption pit formation. Given that osteoclast differentiation and resorptive activity involve a metabolic shift toward glycolysis,^40^ we hypothesized that SphK1 in IDG-CM6 cells modulates glycolysis in osteoclasts. At 48 h post siRNA expression in heavy force-loaded IDG-CM6 cells, CM were collected to culture osteoclasts, which subsequently exhibited a significant decrease in the extracellular acidification rate (ECAR), reflecting reduced glycolysis and glycolytic capacity, compared to osteoclasts cultured with CM from scramble control cells (Fig. 4c). Consistent with this, the elevated levels of specific glycolysis markers, including GLUT1, GLUT3, LDHA, and PKM2^41^ in osteoclasts induced by CM from heavy force-loaded IDG-CM6 cells were partially reversed by SphK1 knockdown (Figure. S9). Similarly, immunofluorescence intensity of GLUT1, LDHA, and PKM2 displayed trends consistent with the expression levels of these markers (Fig. S10). Taken together, these findings indicate that SphK1 plays a significant role in mediating the regulatory effects of heavy force-loaded cementocytes on osteoclastogenesis and osteoclastic glycolytic metabolism.

**Fig. 5:**
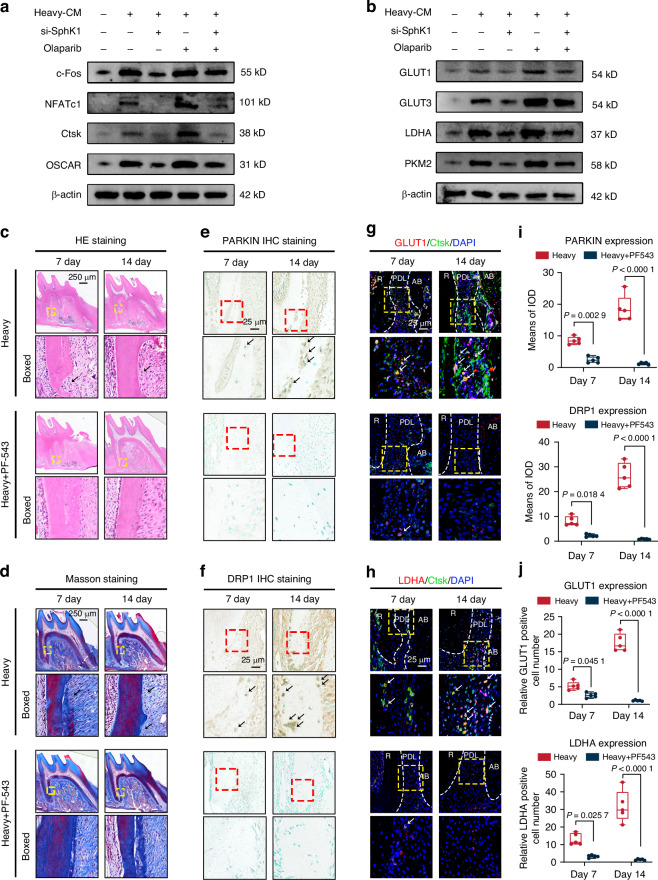
SphK1 inhibition reduces osteoclastic glycolysis and activity as well as cementocyte mitophagy and OIIRR in vivo. **a** The protein expression of c-Fos, NFATc1, Ctsk and OSCAR in BMMs were examined by western blot analysis using GAPDH as a loading control. Quantitative RT-PCR analysis was performed to examine the mRNA levels of *c-Fos*, *Nfatc1*, *Ctsk* and *Oscar*. **b** The protein expression of GLUT1, GLUT3, LDHA and PKM2 in BMMs were examined by western blot analysis using β-actin as a loading control. Quantitative RT-PCR analysis was performed to examine the mRNA levels of *Slc2a1*, *Slc2a3*, *Ldha* and *Pkm*. **c**, **d** Representative HE and Masson’s trichrome staining images of the distobuccal roots of M1 under 100 g orthodontic force at Day 7 and Day 14. PF-543 was adopted to selectively inhibited the expression of SphK1. The black arrow indicated the resorption pits. Scale bar = 250 μm. **e**, **f** Representative immunohistochemical images of PARKIN and DRP1 at the mesial surface of distobuccal roots. The red box indicated the interested area at the lower panel. The black arrow indicated the positive cells at the interested area. Scale bar = 25 μm. **g**, **h** Representative immunofluorescence images of GLUT1 or LDHA (red) at the mesial surface of distobuccal roots. The Ctsk (green) was used to label osteoclasts. The yellow box indicated the interested area at the lower panel. The white arrow indicated the osteoclasts at the interested area. R, Root; PDL, Periodontal ligament; AB, Alveolar bone. Scale bar = 25 μm. **i**, **j** Semi-quantitative analysis of PARKIN and DRP1 positive staining, as well as GLUT1- and LDHA-positive cells, at the interested area. Statistical comparison was performed using two-tailed Student’s *t* test or one-way ANOVA with Tukey’s post hoc test. *P* < 0.05 was considered statistically significant. *n* = 5 for each group. All data were presented as mean ± SEM

To further assess whether SphK1-mediated mitophagy in IDG-CM6 cells regulates osteoclastogenesis and osteoclastic glycolytic metabolism, we used Olaparib, a potent mitophagy agonist,^42^ to promote mitophagy. We observed that the significant reduction in c-Fos, NFATc1, Ctsk, and OSCAR expression caused by CM from si-SphK1-transfected IDG-CM6 cells was partially rescued by Olaparib, indicative of restored osteoclastogenesis (Figs. 5a and S11a). Consistently, the levels of GLUT1, GLUT3, LDHA, and PKM2 followed trends similar to those observed in osteoclastic marker expression (Figs. 5b and S11b). Overall, these results suggest that SphK1-mediated mitophagy in heavy force-loaded IDG-CM6 cells regulates both osteoclastogenesis and osteoclastic glycolytic metabolism.

### SphK1 inhibition reduces cementocyte mitophagy and suppresses OIIRR and osteoclastic glycolysis in vivo

To investigate the role of SphK1 in regulating OIIRR and mitophagy in cementocytes in vivo, PF-543 was administered in an in vivo orthodontic loading model for both 7 and 14 days. Consistent with the arrangement of periodontal fibers observed in Masson’s trichrome staining, the root resorption pits, as detected by HE staining, showed effective repair following PF-543 treatment at both day 7 and day 14 (Fig. 5c, d). Immunohistochemical staining and semi-quantitative analysis revealed a significant reduction in PARKIN- and DRP1-positive staining in the PF-543 group compared to the Heavy group at both time points, indicating that mitophagy in cementocytes induced by heavy force was suppressed (Fig. 5e, f, i). Additionally, osteoclastic glycolytic metabolism and osteoclast formation were evaluated in vivo, and the results demonstrated that SphK1 inhibition significantly reduced the expression of GLUT1 and LDHA in Ctsk-labeled osteoclasts, with their number concomitantly decreased at both day 7 and day 14, suggesting an impairment of SphK1-mediated glycolytic metabolism in osteoclasts during OIIRR (Fig. 5g, h, j). In addition, no significant differences in OTM distance and bone mass were observed between Heavy and SphK1 inhibition group, both at Day 7 and Day 14, during orthodontically induced bone remodeling (Fig. S12).

### Heavy force-induced mitophagy in IDG-CM6 cells drives osteoclastogenesis through mitochondrial transfer

**Fig. 6:**
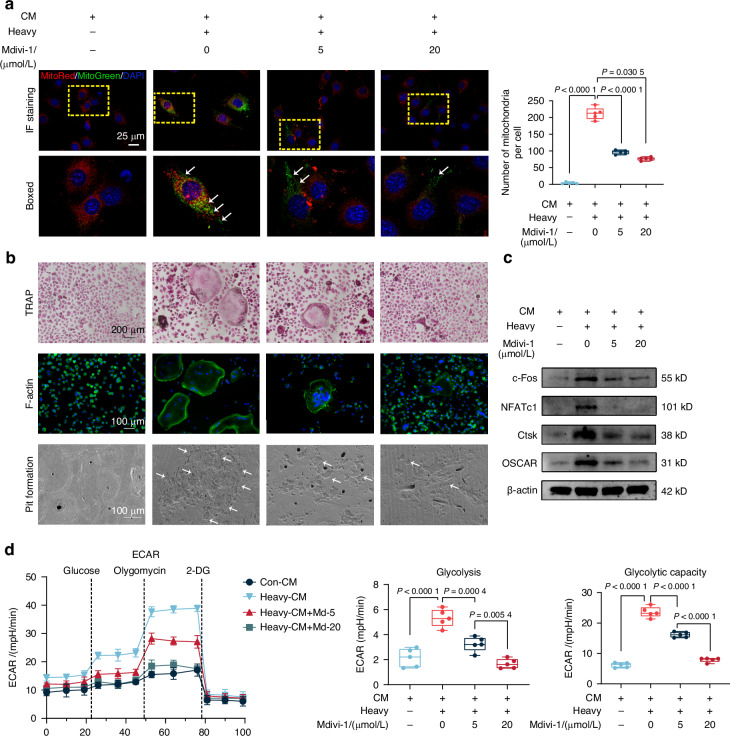
Heavy force-induced mitophagy in IDG-CM6 cells drives osteoclastogenesis through mitochondrial transfer. **a** Representative coimmunostaining images were presented for MitoTracker Green and MitoTracker Deep Red. The yellow box indicated the interested area at the lower panel. The white arrow indicates the mitochondria received from IDG-CM6 cells. Scale bar = 25 μm. **b** Representative images of TRAP staining. The arrows indicated osteoclasts. Scale bar = 200 μm. Representative images of F-actin ring staining. The arrows indicated osteoclasts. Scale bar = 200 μm. The SEM of resorption pits on bovine slices seeded with differentiating osteoclasts were also performed, the white arrows indicated the resorption lacunae on bovine slices. Scale bar = 100 μm. **c** The protein expression of c-Fos, NFATc1, Ctsk and OSCAR in BMMs were examined by western blot analysis using GAPDH as a loading control. **d** The extracellular acidification rate (ECAR) was recorded. The ECAR was normalized to the relative cell counts. Representative ECAR time-course data were shown. Statistical comparison was performed using one-way ANOVA with Tukey’s post hoc test. *P* < 0.05 was considered statistically significant. *n* = 5 for each group. All data were presented as mean ± SEM

**Fig. 7:**
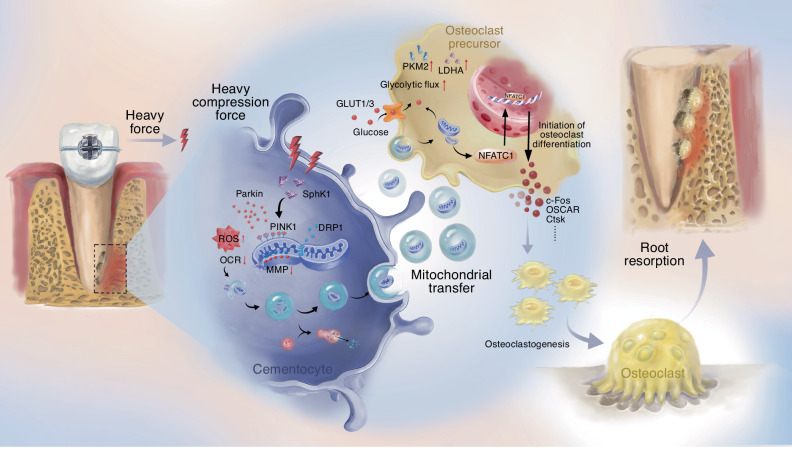
A schematic diagram illustrating the molecular mechanism of SphK1/mitophagy axis in cementocytes driving orthodontic root resorption via mitochondrial transfer to osteoclasts

Previous studies have demonstrated that osteocytes, acting as donor cells, regulate bone metastasis by transferring mitochondria to recipient cells, including osteoclasts and cancer cells.^17,26^ Mitophagy, recognized as a critical mediator of intercellular communication, has been documented in the process of mitochondrial delivery.^23^ Given these findings, we hypothesized that cementocytes, which share functional similarities with osteocytes,^28^ undergo mitophagy in response to heavy force, facilitating the transfer of mitochondria into BMMs to promote osteoclastogenesis. To test this hypothesis, we first assessed mitophagy-mediated mitochondrial transfer from cementocytes to BMMs using Mdivi-1 to inhibit mitophagy in IDG-CM6 cells and subsequently using CM from cementocytes to culture BMMs. Our results demonstrated that heavy force significantly augmented the transfer of MitoTracker Green-labeled mitochondria from IDG-CM6 cells to BMMs, an effect that was partially attenuated in a dose-dependent manner by Mdivi-1 (Fig. 6a).^20^ In addition, to obtain direct genetic evidence for mitochondrial transfer, we employed a species-specific mtDNA tracing assay. Human THP-1 macrophages were cultured with CM from mouse IDG-CM6 cementocytes. PCR analysis using species-specific primers revealed that THP-1 cells incubated with CM from heavy force-loaded IDG-CM6 cells contained a detectable level of mouse mtDNA, which was significantly higher than that in cells treated with CM from unloaded controls (Fig. S13). This increase in heterospecific mtDNA was abolished when the donor cementocytes were pre-treated with the mitophagy inhibitor Mdivi-1, in a dose-dependent manner, demonstrating the dependency of mitochondrial transfer on the mitophagic machinery. To further elucidate the role of mitophagy-induced mitochondrial transfer in osteoclastogenesis, we used CM from IDG-CM6 cells for in vitro osteoclastogenesis assays. As depicted in Figs. 6b and S14a, the increase in osteoclast formation and resorption pits induced by Heavy-CM was significantly reversed when cells were cultured in CM from IDG-CM6 cells treated with Mdivi-1, in a dose-dependent manner. Consistently, the expression of osteoclast-specific markers, including c-Fos, NFATc1, Ctsk, and OSCAR, was elevated in the Heavy-CM group, compared to the Con-CM group, but diminished in the Mdivi-1 treatment group in a dose-dependent manner (Figs. 6c and S14b). Furthermore, similar trends in ECAR, as well as glycolysis and glycolytic capacity based on ECAR measurements, were also observed in Heavy-CM-treated BMMs with administration of Mdivi-1 (Fig. 6d). Taken together, these findings suggest that heavy force-induced mitophagy in cementocytes enhances osteoclastogenesis and osteoclastic glycolysis through mitochondrial transfer (Fig. 7).

## Discussion

Here, we identified a pivotal role for SphK1 in osteoclast-mediated OIIRR through the regulation of mitophagy-driven mitochondrial transfer from heavy force-loaded cementocytes to osteoclasts. SphK1 enhanced mitophagy in heavy force-loaded cementocytes, which in turn facilitated osteoclastogenesis, osteoclastic glycolytic metabolism, and ultimately contributed to OIIRR. Moreover, we underscored the significance of mitophagy in heavy force-loaded cementocytes in modulating osteoclastogenesis by mediating the transfer of mitochondria to osteoclasts.

In this study, we used light force and heavy force to respectively simulate two distinct scenarios typically encountered during orthodontic treatment. During OTM, root resorption is little under light force, while heavy force frequently leads to significant alveolar bone and root resorption.^6^ Although cementoblasts are actively involved in the repair of the root surface,^14^ cementocytes play a crucial role in root resorption induced by heavy orthodontic force.^7,15,16,43^ Specifically, heavy force upregulates the RANKL/OPG ratio while downregulating OPN level, both within cementocytes, thereby promoting a catabolic response in the root cementum.^16,43^ Recent research has increasingly highlighted the regulatory role of cementocytes in osteoclastogenesis, a key process driving both alveolar bone and root resorption.^13,15,16^ De Rossi et al. reported that cementocytes facilitated osteoclast recruitment and root resorption under external inflammatory stimuli.^15^ Building on this, our previous study demonstrated that cementocyte-osteoclast communication is the central mechanism of OIIRR, with the SphK1/S1P axis serving as a critical mediator in this process.^7^ However, while the mechanism underlying heavy force-induced cementocyte-osteoclast communication has been investigated, it remains incompletely understood and requires further elucidation. In this study, we observed that heavy orthodontic force induced OIIRR characterized by an increase in the number of osteoclasts adhered to the root surface, accompanied by an upregulation of mitophagy-related markers in cementocytes. Consequently, we hypothesize that mitophagy might be actively involved in cementocyte-osteoclast communication, thereby mediating OIIRR.

Mitophagy, the selective autophagy of mitochondria, targets dysfunctional mitochondria for degradation via receptor-mediated mechanisms.^44^ While mitophagy operates as a routine homeostatic process under physiological conditions, it can be markedly upregulated in response to pathological stress, where it often functions as an adaptive, protective response.^45^ In response to external stimuli such as cellular damage, nutrient deprivation, or oxidative stress, mitochondria may become depolarized, lose membrane potential, and subsequently trigger mitophagy.^46,47^ Notably, mechanical cues have recently been identified as a critical inducer of mitophagy.^32,48–51^ However, the effects of mechanical force on mitophagy are highly context-dependent, with varying magnitudes of force exerting distinct influences.^49^ For instance, excessive mechanical load has been shown to impair mitophagy and contribute to cartilage degradation, whereas moderate mechanical force promotes chondrogenesis.^49^ Contrary to this finding, our results demonstrate that mitophagy in cementocytes remains unaltered under light force but is significantly enhanced under heavy force. This divergence may be attributed to differing loading methodologies employed in these studies. While the previous study utilized dynamic compressive loading, our study applied static compression force, which more closely mimics the characteristics of orthodontic force,^7,52^ thereby provoking a more physiologically relevant cellular response. Notably, excessive force may trigger adaptive responses beneficial to the organism,^51^ as evidenced by the upregulation of mitophagy, which accelerates mitochondrial turnover, thereby enabling cells to adapt to the overloaded mechanical stress. In line with our findings, recent research has confirmed that compression force promoted PINK1/PARKIN-dependent mitophagy in periodontal ligament stem cells (PDLSCs).^48^ Given the established role of SphK1 as a key mechanotransducer in cementocytes and emerging evidence linking SphK1/S1P signaling to the regulation of mitochondrial fission and mitophagy, through modulating DRP1 recruitment on mitochondria, in other cell types,^38,53^ we hypothesized that SphK1 might serve as the critical link converting mechanical stress into a mitophagic response in cementocytes. Thus, we investigated the key regulatory role of SphK1 in heavy force-induced mitophagy. Our preliminary study revealed a significant upregulation of SphK1 in cementocytes under excessive mechanical loading,^7,13^ consistent with the trend of mitophagy observed in this study. Based on this, we used the SphK1-specific inhibitor PF-543 and siRNA to knock down SphK1 expression and demonstrated that heavy force enhanced mitophagy in cementocytes through the upregulation of SphK1. Our loss- and gain-of-function data definitively establish SphK1 as a necessary regulator, which bridges our previous work on SphK1-mediated mechanotransduction with the novel mitophagy-driven communication pathway described here. Consistently, previous studies have shown that SphK1 promotes mitochondrial fission,^53^ a process closely linked to mitophagy, and facilitates PINK1-p62 mediated mitophagy.^54^

Beyond its canonical role in intracellular quality control, mitophagy is increasingly recognized as a pivotal mechanism in intercellular communication, particularly through the facilitation of mitochondrial transfer.^23,24^ Our findings that SphK1-mediated mitophagy in mechanically-stimulated cementocytes critically regulates osteoclast differentiation and glycolytic metabolism prompted the hypothesis that mitophagy might orchestrate an outward signaling event. This notion is supported by our prior work confirming the capacity of force-loaded cementocytes to dictate osteoclast biological activity,^7^ leading us to speculate that mitophagy may trigger the extracellular release of signaling entities. Intriguingly, organelles themselves have emerged as potent signaling units in paracrine and juxtacrine signaling. As a fundamental modality of mitochondrial quality control,^55^ mitophagy has been linked to the packaging of mitochondria for intercellular delivery. Various studies have demonstrated that extracellular vesicles are crucial for the transmission of mitochondria and components, effectively transporting functional organelles to recipient cells to enhance their bioenergetics and functional capacity.^18,23,55,56^ Specifically, a seminal study demonstrated that mesenchymal stem cells employ mitophagy to package mitochondria into arrestin domain-containing protein 1-mediated microvesicles (ARMMs), which are subsequently unloaded and engulfed by macrophages, leading to enhanced bioenergetics in the latter.^23^ Building on this paradigm, we sought to determine whether cementocyte mitophagy facilitates mitochondrial donation to osteoclast precursors. Our results robustly demonstrate that heavy force promotes the transfer of mitochondria from IDG-CM6 cells to OCPs, an effect that is dose-dependently abolished by the mitophagy inhibitor Mdivi-1. This finding positions mitophagy not merely as a degradative pathway but as a proactive mechanism enabling cementocytes to supply metabolic substrates to osteoclasts. Consequently, the enhanced osteoclastogenesis and glycolytic flux observed under heavy force can be directly attributed to the acquisition of functional mitochondria from the cementocyte niche.

While our findings highlight SphK1 inhibition as a promising strategy to mitigate OIIRR, a pertinent clinical consideration is the potential effect on cementocyte viability. Our in vitro data show that SphK1 inhibition under heavy force alters mitochondrial parameters, which could theoretically predispose cells to stress. However, the net in vivo outcome was a clear preservation of root structure. This suggests that the primary therapeutic benefit of SphK1 inhibition in this context may stem from disrupting the pro-osteoclastogenic signal (mitochondrial transfer) rather than directly promoting cementocyte survival. Importantly, by preventing excessive mitophagic activation, such inhibition might help maintain cellular defense capacity.^57^ Evidence shows that overactive PINK1/PARKIN-mediated mitophagy can deplete p62 and suppress the Keap1-Nrf2 cytoprotective pathway, thereby amplifying injury.^57^ Thus, moderating mitophagic flux via SphK1 inhibition could paradoxically sustain endogenous antioxidant responses, offsetting potential negative effects on cell survival. Future therapeutic development should aim for localized and transient modulation of this axis, seeking a dosage that effectively uncouples mechanotransduction from resorptive signaling while preserving cellular homeostasis.

In summary, our study uncovers a previously unrecognized mechanotransduction pathway in which SphK1 acts as a central hub, converting excessive orthodontic force into a metabolic signal that fuels osteoclastogenesis via mitophagy-driven mitochondrial transfer. We demonstrate that heavy force triggers SphK1-dependent mitophagy in cementocytes, a process that is not merely adaptive for intracellular clearance but is fundamentally communicative. The subsequent intercellular transfer of mitochondria from cementocytes to osteoclast precursors serves as a critical metabolic subsidy, enhancing glycolytic flux and empowering the resorptive capacity of osteoclasts, thereby driving OIIRR progression. This SphK1-mitophagy-transfer axis redefines our understanding of cementocyte-osteoclast communication, positioning cementocytes not as passive victims but as active metabolic regulators of root resorption. Our work thus identifies SphK1-mediated mitophagy and the mitochondrial transfer process as key mechanistic links and novel therapeutic targets, thereby revealing a promising strategy to prevent OIIRR.

## Materials and methods

### In vivo orthodontic loading

All studies used 6-week-old male Wistar rats under a protocol approved by the Ethics Committee of West China Hospital of Stomatology (WCHSIRB-D-2024-204), adhering to institutional guidelines and ARRIVE standards. Rats (*n* = 5 per group) were randomly assigned to Control, Light, or Heavy force groups. Orthodontic tooth movement was achieved using a nickel-titanium closed-coil spring (Grikin Advanced Materials) delivering 0 g, 25 g, or 100 g of force between the maxillary left first molar (M1) and incisors.^7^ At Day 14, alveolar bone specimens containing M1 were collected after euthanasia by pentobarbital overdose for subsequent analysis.

To assess the therapeutic efficacy of SphK1 inhibition, an additional cohort (*n* = 10/group) received either vehicle or local gingival injections of PF-543 (0.5 mg/kg every other day; MCE), a selective SphK1 inhibitor, concurrent with orthodontic force application.^7,53^ Euthanasia and tissue collection were performed at Day 7 and 14. Throughout the experiments, animals were monitored twice daily, and orthodontic appliances were checked every 48 h and repaired immediately if damaged. All alveolar bone blocks that included M1 were harvested and fixed immediately in 10% formalin for further analysis.

### Microcomputed tomography (micro-CT) analysis

High-resolution microtomographic imaging was performed on all specimens using a Scanco Medical μCT 50 system. Volumetric data were acquired at a 10 μm isotropic resolution and subjected to noise reduction with a 3D Gaussian filter (mean = 1.2). Digital reconstruction of alveolar bone architecture was conducted in Mimics 21.0 software employing a standardized mineral density threshold (3 000 Hounsfield units). In the animal experiments conducted in this study, since orthodontic traction force was applied in a single direction, compression force was produced and loaded mainly on a specific root surface, that is, the mesial surface. Thus, we speculated that root resorption primarily occurred on the mesial aspect of the root. Quantification of root resorption focused on the mesial aspect of the distobuccal root, the primary compression zone during orthodontic loading, with the unaffected contralateral molar serving as an intrinsic control.^7^ After three-dimensional alignment along standardized anatomical planes, computational segmentation of roots at the furcation point was performed using 3-matic Research 13.0, allowing for precise volumetric quantification of resorptive pits relative to contralateral morphology.^7^

The amount of OTM was measured by the spacing between the cementum-enamel junction (CEJ) levels of the first and second left molars. In this study, a 200 µm × 200 µm × 600 µm cube of trabecular bone mesial to the middle part of the distobuccal root of the maxillary left first molar was selected as the region of interest for analysis. The distance between the cube and the root was 100 µm. Then, parameters including the bone volume/total volume (BV/TV) ratio, trabecular number (Tb.N) and trabecular spacing (Tb.Sp) were calculated at day 7 and day 14 after OTM.

### Histological and immunohistochemical staining

Following micro-CT analysis, specimens were sequentially processed through 24-h fixation in 10% neutral buffered formalin, decalcification in 14% EDTA (pH 7.1), paraffin embedding, and sectioning at 5 μm thickness. Consecutive sections were stained with hematoxylin and eosin (Solarbio) and Masson’s trichrome (G1340, Solarbio) using established protocols, then resin-mounted for light microscopic examination (Leica). The mesial aspect of distobuccal roots was defined as the region of interest.^7^ Osteoclast activity was assessed by TRAP staining (387A Kit, Sigma-Aldrich), with multinucleated positive cells quantified along the compression surface and normalized per millimeter of root length. For immunohistochemistry, sections were incubated overnight at 4 °C with primary antibodies against PARKIN (1:200, Proteintech) and LC3B (1:200, Proteintech), followed by a 30-min incubation at 37 °C with appropriate HRP-conjugated secondary antibodies (1:1 000). All histological quantifications were performed in a blinded manner using Image-Pro Plus 6.0 software.

### Immunofluorescence staining (tissue)

Tissue sections from the compressed distobuccal root region were processed for immunofluorescence to detect NFATc1, GLUT1, LDHA, and DRP1 expression in osteoclasts. Following antigen retrieval in citrate buffer (95 °C, 30 min) and blocking with 10% goat serum (37 °C, 30 min), sections were incubated overnight at 4 °C with primary antibodies against NFATc1 (1:100, ABclonal), GLUT1 (1:500, Proteintech), or LDHA (1:100, ABclonal), followed by co-staining with an osteoclast-specific marker antibody against Ctsk (1:400, Proteintech). Parallel staining for DRP1 (1:500, Proteintech) was performed under identical conditions. All sections were then incubated with species-specific Alexa Fluor-conjugated secondary antibodies (594 anti-rabbit and 488 anti-mouse, 1:200; Abcam) for 2 h at 37 °C. After nuclear counterstaining with DAPI, images were captured using a Leica DMI 6000 fluorescence microscope. Osteoclasts positive for NFATc1, GLUT1, or LDHA along the compression surface were quantified per 50 μm root length.

### Cell Culture

IDG-CM6 cells were maintained in an undifferentiated state with 50 μg/mL IFN-γ (Gibco) at 33 °C. Upon reaching 80% confluence within 2 days, the cells were induced to differentiate by switching to a medium containing 50 μg/mL ascorbic acid and 4 mmol/L β-glycerophosphate, without IFN-γ, and were then cultured at 37 °C for 21 days prior to mechanical loading.

For bone marrow-derived macrophages (BMMs), bone marrow cells were isolated from the femurs and tibias of 6- to 8-week-old C57BL/6 mice. After 16 h of initial culture, the non-adherent cell population was collected and seeded onto culture plates. These cells were then expanded for 2 days in the presence of 80 ng/mL macrophage colony-stimulating factor (M-CSF; Hangzhou Yangming Biotechnology Co. Ltd).^7^

For THP-1 cells, a human monocytic cell line, were seeded at a concentration of 1 × 10^5^ cells/well. Prior to seeding, the cells were stimulated with phorbol myristate acetate (PMA, 50 ng/mL; Sigma) for 72 h.

### In vitro mechanical loading

Prior to mechanical loading, the culture medium was replaced with serum-free α-MEM. A static compressive force was then applied to confluent IDG-CM6 cells using a previously established method.^7^ Briefly, a glass cylinder was placed over the cell layer, and force magnitudes of 0.5 g/cm^2^ (light) or 3.0 g/cm^2^ (heavy) were achieved by adding lead granules. Unloaded cells served as controls. Cells were analyzed 6 h post loading. Conditioned media (CM) were collected, centrifuged at 3 000 r/min for 10 min to remove cellular debris, and the resulting supernatant was used immediately for subsequent experiments.

### Cell treatment and transfection

To investigate the role of SphK1 in cementocyte mitophagy, IDG-CM6 cells were subjected to pharmacological or genetic interventions prior to mechanical loading. For pharmacological inhibition, cells were pretreated for 24 h with PF-543 (10, 50, or 200 nmol/L; MCE) or an equivalent volume of DMSO as a vehicle control. For genetic knockdown, cells were transfected with SphK1-targeting siRNAs (Hanheng Biotechnology) or scrambled controls using Lipofectamine 3000 (Invitrogen). To modulate mitophagy directly, cells were either pretreated with 5 μmol/L Olaparib for 24 h to induce mitophagy or with 20 μmol/L Mdivi-1 for 2 h to inhibit it,^32^ with DMSO serving as the vehicle control in both cases.

### RNA sequencing and data analysis

Total RNA was extracted from IDG-CM6 cells subjected to heavy (3.0 g/cm^2^) compressive force for 6 h and subsequently processed for RNA sequencing on an Illumina HiSeq 2500 platform (Novogene). De novo transcriptome assembly was performed using Trinity, and clean reads from each sample were aligned to the reference sequence via RSEM. Transcript clustering was conducted with Corset, and gene expression levels were normalized using DESeq2. Genes with an adjusted *P*-value < 0.05 were defined as differentially expressed genes (DEGs). DEG-based heatmaps were generated by k-means clustering (Euclidean distance) and visualized in Java TreeView. Gene ontology (GO) enrichment analysis for DEGs was carried out using the DAVID online database, with the most significant terms selected based on *P* values.

### Reactive oxygen species (ROS)

Intracellular and mitochondrial ROS levels were assessed using fluorescent probes. Cells were incubated with 10 μmol/L 2′,7′-dichlorofluorescein diacetate (DCFH-DA, Beyotime) and Hoechst 33342 (Beyotime) in α-MEM for 20 min at 37 °C to detect total intracellular ROS. Mitochondrial ROS (mtROS) was specifically measured using 5 μmol/L MitoSOX Red (Yeasen) for 10 min at 37 °C. Fluorescent signals were captured by confocal laser scanning microscopy (Olympus).

### Mitochondrial membrane potential (MMP)

Mitochondrial membrane potential (MMP) was assessed using the JC-1 assay kit (Beyotime). Following a 20-min incubation with JC-1 staining solution at 37 °C, cells were imaged by confocal microscopy (Olympus). The JC-1 probe forms red-fluorescent aggregates in mitochondria with intact membrane potential but converts to green-fluorescent monomers upon depolarization.

### Transmission electron microscope (TEM)

For transmission electron microscopy (TEM) analysis, cells were sequentially fixed with 2.5% glutaraldehyde and post-fixed in 1% osmium tetroxide. Following dehydration through a graded ethanol series, samples were embedded in epoxy resin. Ultrathin sections (70–90 nm) were prepared and double-stained with 2% uranyl acetate and 3% lead citrate. Subcellular ultrastructure was then visualized using a JEM-1200 EX transmission electron microscope (JEOL) at an accelerating voltage of 80 kV.

### Metabolic flux analysis

Cellular bioenergetics were profiled using a Seahorse XFe96 Analyzer (Agilent). Cells were seeded at a density of 1 × 10⁴ cells per well in Cell-Tak-coated XF96 microplates, with pre-assay density and viability confirmed using a Cytation-1 imaging reader (BioTek). Glycolytic function was assessed by measuring the extracellular acidification rate (ECAR) following sequential injection of 10 mmol/L glucose, 1 μmol/L oligomycin, and 50 mmol/L 2-deoxyglucose (2-DG) to determine basal glycolysis, glycolytic capacity, and glycolytic reserve, respectively. Mitochondrial respiratory function was evaluated via the oxygen consumption rate (OCR) following sequential addition of 1 μmol/L oligomycin, 1 μmol/L FCCP, and 0.5 μmol/L rotenone/antimycin A to quantify ATP-linked, maximal, and non-mitochondrial respiration. All data were normalized to total protein content and analyzed using Wave Software 2.6.1 (Agilent), with OCR and ECAR expressed as pmol/min/μg and mpH/min/μg, respectively.

### Western blot

Protein samples were separated by SDS-PAGE and transferred onto PVDF membranes. After blocking with 5% BSA, the membranes were incubated overnight at 4 °C with the following primary antibodies: PINK1 (1:1 000, Proteintech 23274-1-AP), PARKIN (1:1 000, Proteintech 14060-1-AP), DRP1 (1:2 000, Proteintech 12957-1-AP), FIS1 (1:2 000, Proteintech 10956-1-AP), c-Fos (1:1 000, CST 31254T), NFATc1 (1:1 000, ABclonal A1539), Ctsk (1:1 000, Proteintech 11239-1-AP), OSCAR (1:1 000, Huabio ER61888), GLUT1 (1:1 000, Proteintech 21829-1-AP), GLUT3 (1:3 000, Proteintech 20403-1-AP), LDHA (1:1 000, ABclonal A1146), PKM2 (1:1 000, ABclonal A20991). GAPDH (1:10 000, Huabio EM1101) and β-actin (1:20 000, Proteintech 66009-1-Ig) were used as loading controls. Following incubation with HRP-conjugated secondary antibodies (ZSGB-Bio) for 1 h at room temperature, signals were developed using Clarity™ Western ECL Substrate (Bio-Rad) and visualized with a ChemiDoc Touch Imaging System (Bio-Rad). Band intensities were quantified using ImageJ software (v1.51) after normalization to loading controls.

### Quantitative real-time reverse transcription polymerase chain reaction (qRT-PCR)

Total RNA was isolated with TRIzol reagent (Invitrogen) and quantified spectrophotometrically. Complementary DNA was synthesized using the SuperScript III First-Strand Synthesis System (Invitrogen). Quantitative real-time PCR was then performed on a QuantStudio 3 system (Thermo Fisher) with TB Green Premix Ex Taq II (Takara). Gene expression levels were normalized to Gapdh or Actb and calculated by the 2^–ΔΔCt^ method. All primer sequences are provided in Appendix Table 1.

### Immunofluorescence staining (cell)

For F-actin ring staining, BMMs (1 × 10⁵ cells/well) were differentiated under osteoclastogenic conditions, then fixed with 4% PFA and permeabilized with 0.5% Triton X-100. After blocking with 5% BSA, F-actin was labeled with phalloidin (6 μmol/L, Invitrogen) and nuclei were counterstained with DAPI.

For immunofluorescence, cells were incubated overnight at 4 °C with the following primary antibodies: NFATc1 (ABclonal A1539, 1:100), LAMP1 (Abcam ab24170, 1:500), TOMM20 (Abcam ab56783, 1:200), GLUT1 (Proteintech 21829-1-AP, 1:300), LDHA (ABclonal A1146, 1:100), PKM2 (ABclonal A20991, 1:100), PINK1 (Proteintech 23274-1-AP, 1:500), PARKIN (Proteintech 14060-1-AP, 1:500), DRP1 (Proteintech 12957-1-AP, 1:500), FIS1 (Proteintech 10956-1-AP, 1:500), ATG5 (Proteintech 10181-2-AP, 1:200), ATG7 (Proteintech 10088-2-AP, 1:300), Beclin 1 (Proteintech 11306-1-AP, 1:500), LC3B (Proteintech 14600-1-AP, 1:500). This was followed by a 2-h incubation with species-matched Alexa Fluor-conjugated secondary antibodies (1:200, Abcam) at room temperature.

All images were acquired using either fluorescence microscopy (Leica) or confocal laser scanning microscopy (Olympus) and analyzed with ImageJ software (NIH).

### Mitochondria transfer assay

To assess mitochondrial transfer,^56^ IDG-CM6 cells and BMMs were pre-labeled with MitoTracker Green (50 nmol/L) and MitoTracker Deep Red (50 nmol/L), respectively. BMMs were then cultured for 24 h in conditioned medium collected from mechanically loaded IDG-CM6 cells. The transfer of mitochondria from IDG-CM6 cells (green) to BMMs (red) was visualized by confocal laser scanning microscopy (Olympus).

### Mitochondrial DNA transfer analysis

To provide genetic evidence for mitochondrial transfer, a species-specific mtDNA detection assay was performed. Conditioned media from mouse IDG-CM6 cells (subjected to heavy force or control conditions, with or without Mdivi-1 pretreatment) were used to culture human THP-1-derived macrophages for 24 h. mtDNA was isolated from cells using the Mitochondrial DNA Isolation Kit (Abcam, ab65321). The hypervariable region (HVR) of mitochondrial DNA was amplified by PCR using species-specific primers. The mouse-specific primer pair was: forward, 5′- CAGCCCATGACCAACATAACT-3′; reverse, 5′-GGACTAATGATTCTTCACCGTAGG-3′ (amplicon: 137 bp). The human-specific primer pair was: forward, 5′-CGCCCACTAGGATACCAACA-3′; reverse, 5′-GGACGAGAAGGGATTTGACTACA-3′ (amplicon: 101 bp). PCR products were analyzed by agarose gel electrophoresis. The intensity of the mouse-specific band was quantified using ImageJ software and expressed as a percentage of the total (mouse + human) mtDNA signal from the same sample.

### Statistical analysis

All statistical analyses were conducted using SPSS 22.0 (IBM). Data are presented as mean ± SD in line graphs or as boxplots displaying the median, interquartile range, and full data range, with individual data points overlaid. Group comparisons were performed using two-tailed Student’s *t*-tests or one-way ANOVA with Tukey’s post hoc test. A *P*-value of less than 0.05 was considered statistically significant. All experiments were repeated at least five times independently.

## Supplementary information

Appendix
