# Supplementary material for: SphK1/mitophagy axis in cementocytes drives orthodontic root resorption via mitochondrial transfer to osteoclasts
Source: Bone Res. 2026 May 14;14:52. doi: 10.1038/s41413-026-00538-0 (PMC13176349; doi:10.1038/s41413-026-00538-0)
Supplement: Supplementary file 1 — Appendix [file 41413_2026_538_MOESM1_ESM.docx]

**SphK1/mitophagy axis in cementocytes drives orthodontic root resorption via mitochondrial transfer to osteoclasts**

Han Wang^1,2,3*^, Sihang Chen^2,3*^, Shuo Chen^1^, Chengchen Duan^1^, Li Zhu^1^, Hengyi Lin^1^, Shujuan Zou^1^, Yu Li^1^, Peipei Duan^1^

**Appendix**

**Appendix Figure**


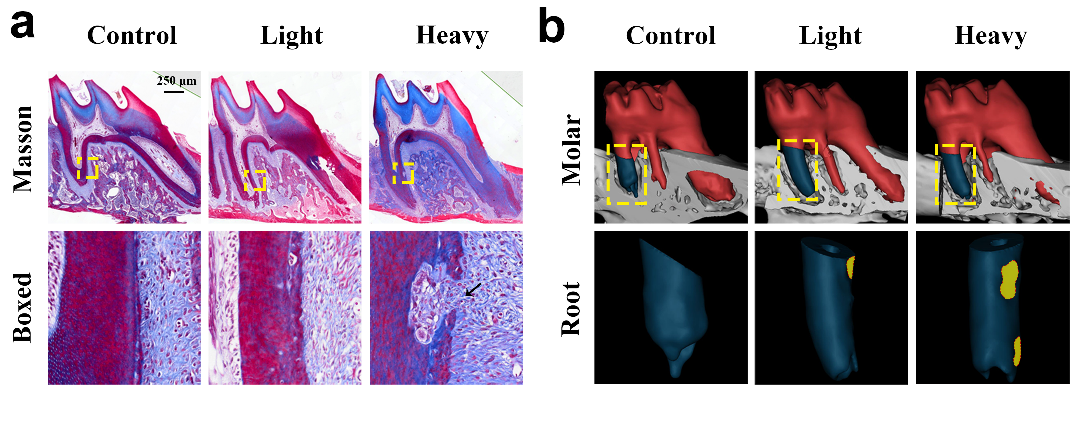


**Appendix Figure 1.** Heavy orthodontic force induces OIIRR. **a** Representative Masson’s trichrome staining images of the distobuccal roots of M1 under 100 g orthodontic force at Day 14. The black arrow indicated the resorption pits. Scale bar = 250 μm. **b** The distobuccal roots of M1 were scanned by microcomputed tomography and reconstructed to calculate the root resorption volumes using Mimics 21.0. The yellow box indicated the distobuccal root of M1. The yellow area indicated the resorption pits.


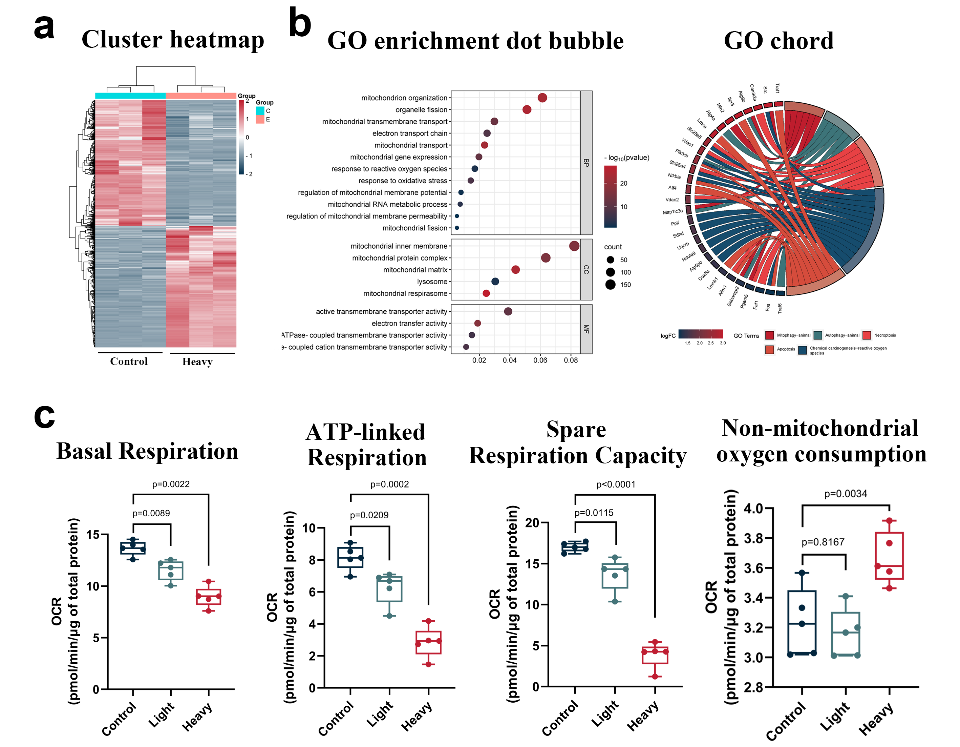


**Appendix Figure 2.** The RNA-seq of IDG-CM6 cells under heavy compression force and the oxygen consumption rate (OCR)-related analyses. **a** The heatmap summarized the significantly expressed genes between the Control and Heavy group. **b** Gene Ontology (GO) enrichment analysis of differentially expressed genes (>2.0-fold) showing significantly enriched processes related to mitochondrial bioenergetics and dynamics. **c** The associated data based on the OCR were shown as basal respiration, ATP-linked respiration, and Spare respiration capacity and non-mitochondrial oxygen consumption. Statistical comparison was performed using one-way ANOVA with Tukey’s post hoc test. p < 0.05 was considered statistically significant. n = 5 for each group. All data were presented as mean ± SEM.


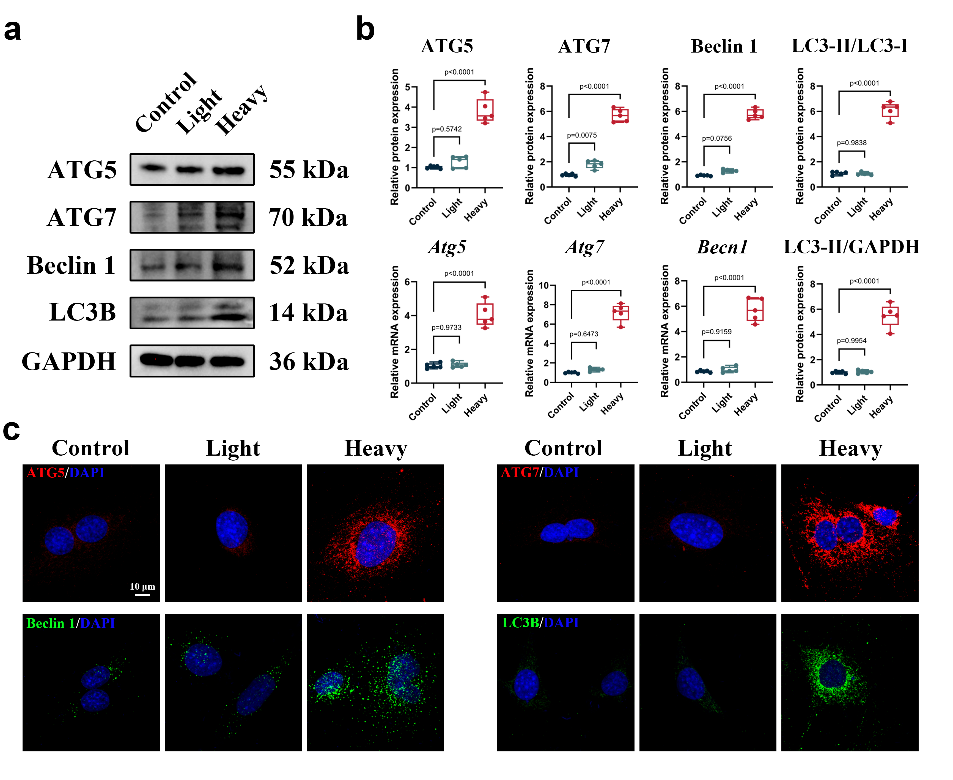


**Appendix Figure 3.** The autophagy level in IDG-CM6 cells under light (0.5 g/cm^2^) or heavy compression force (3.0 g/cm^2^). **a** The protein expression of ATG5, ATG7, Beclin1, and LC3B in IDG-CM6 cells under light or heavy compression force were assessed by western blot analysis using GAPDH as a loading control. **b** The Quantitative analysis of western blot was performed to examine the protein levels of ATG5, ATG7, Beclin1, LC3B-II and LC3B-II/I, and quantitative RT-PCR analysis was performed to examine the mRNA levels of *Atg5*, *Atg7* and *Becn1* in IDG-CM6 cells. The Representative immunofluorescence images of ATG5, ATG7, Beclin1, LC3B in IDG-CM6 cells under light or heavy force. Scale bar = 10 μm. Statistical comparison was performed using one-way ANOVA with Tukey’s post hoc test. p < 0.05 was considered statistically significant. n = 5 for each group. All data were presented as mean ± SEM.


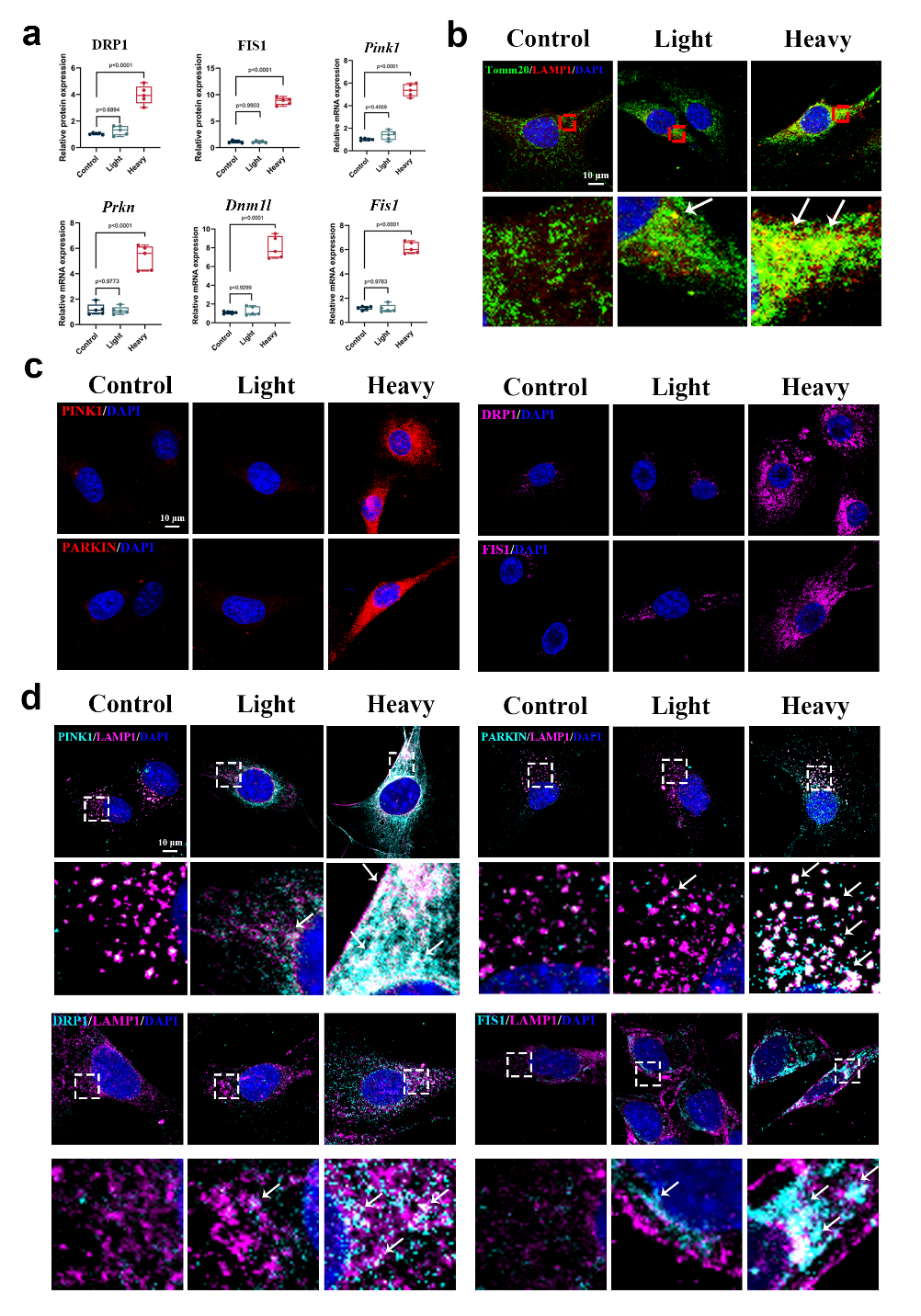


**Appendix Figure 4.** The protein and mRNA expressions of mitophagy-related markers and coimmunostaining images of mitochondria with lysosomes. **a** The quantitative analysis of protein expression of DRP1 and FIS1 was performed. Quantitative RT-PCR analysis was performed to examine the mRNA levels of *Pink1*, *Prkn*, *Dnm1l* and *Fis1*. **b** Representative coimmunostaining images were presented for Tomm20 with LAMP1. The red box indicated the interested area at the lower panel. The white arrow indicates the colocation of mitochondria and lysosomes. Scale bar = 10 μm. **c** The Representative immunofluorescence images of PINK1, PARKIN, DRP1 and FIS1 in IDG-CM6 cells under light or heavy force. Scale bar = 10 μm. **d** Representative coimmunostaining images were presented for PINK1, PARKIN, DRP1 and FIS1, with LAMP1. The white box indicated the interested area at the lower panel. The white arrow indicated the colocalization of PINK1, PARKIN, DRP1 and FIS1 with lysosomes, respectively. Scale bar = 10 μm. Statistical comparison was performed using one-way ANOVA with Tukey’s post hoc test. p < 0.05 was considered statistically significant. n = 5 for each group. All data were presented as mean ± SEM.


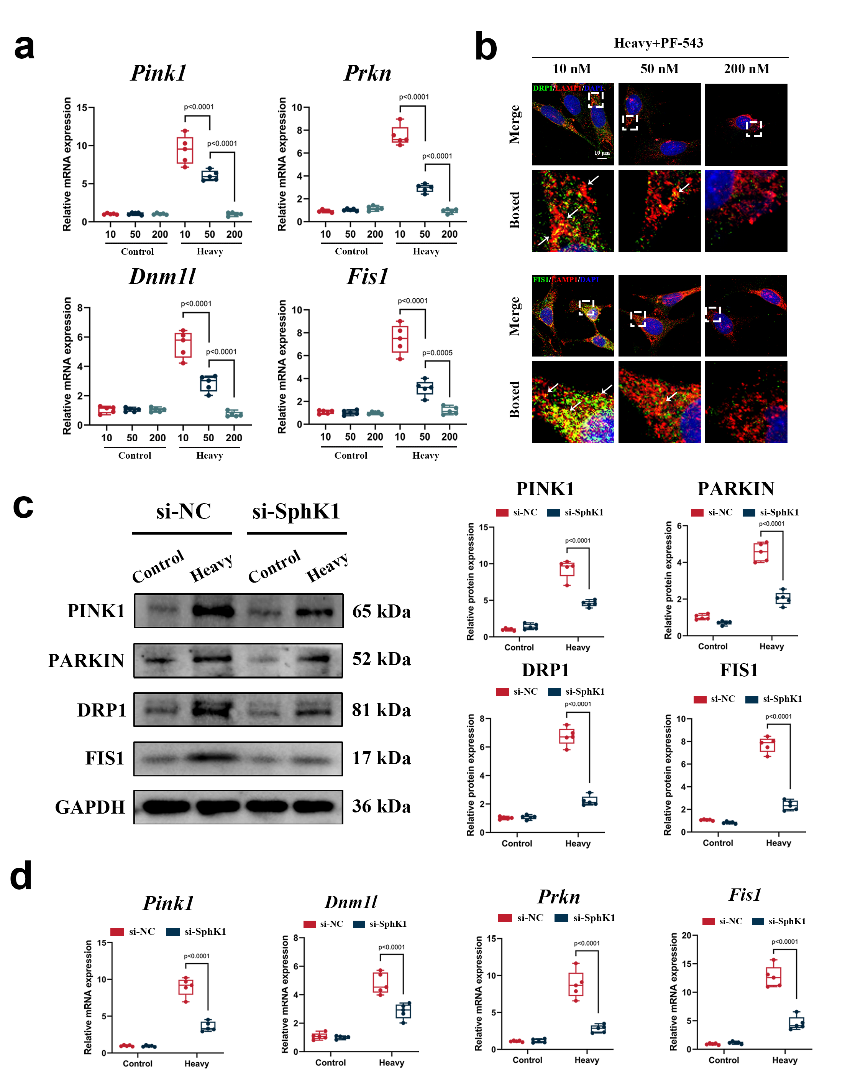


**Appendix Figure 5.** **a** The Quantitative RT-PCR analysis was performed to examine the mRNA levels of *Pink1*, *Prkn*, *Dnm1l* and *Fis1*. **b** Representative coimmunostaining images were presented for DRP1 and FIS1, with LAMP1. The white box indicated the interested area at the lower panel. The white arrow indicated the colocalization of PINK1, PARKIN, DRP1 and FIS1 with lysosomes, respectively. Scale bar = 10 μm. **c, d** The protein expression of PINK1, PARKIN, DRP1 and FIS1 were examined by western blot analysis using GAPDH as a loading control. The quantitative analysis of protein expression of PINK1, PARKIN, DRP1 and FIS1 was also performed. Quantitative RT-PCR analysis was performed to examine the mRNA levels of *Pink1*, *Prkn*, *Dnm1l* and *Fis1*. Statistical comparison was performed using two-tailed Student’s t test or one-way ANOVA with Tukey’s post hoc test. p < 0.05 was considered statistically significant. n = 5 for each group. All data were presented as mean ± SEM.


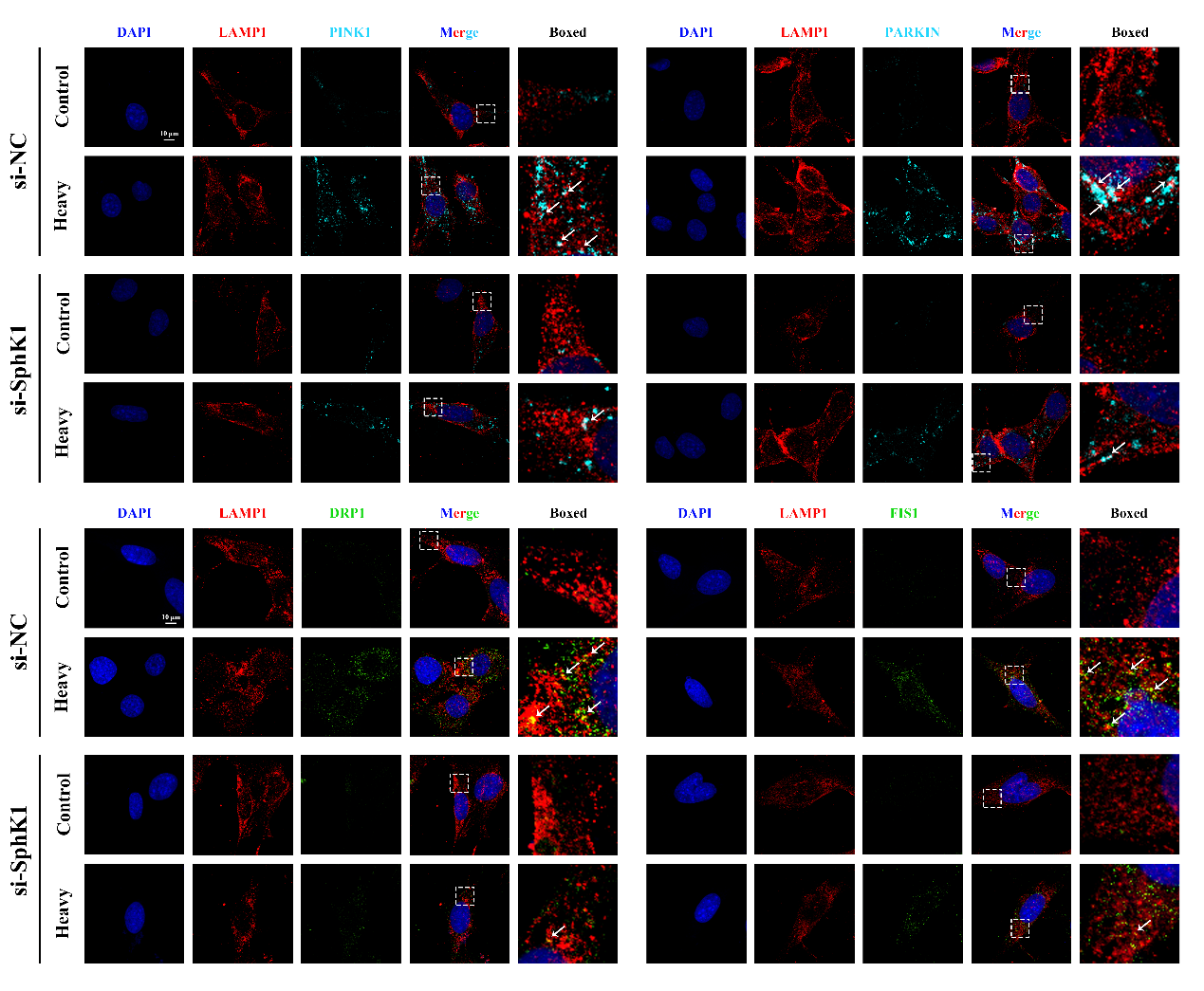


**Appendix Figure 6.** The immunofluorescence and colocalization of PINK1, PARKIN, DRP1 and FIS1 with lysosomes, after si-SphK1 transfected in IDG-CM6 cells. Scale bar = 10 μm.


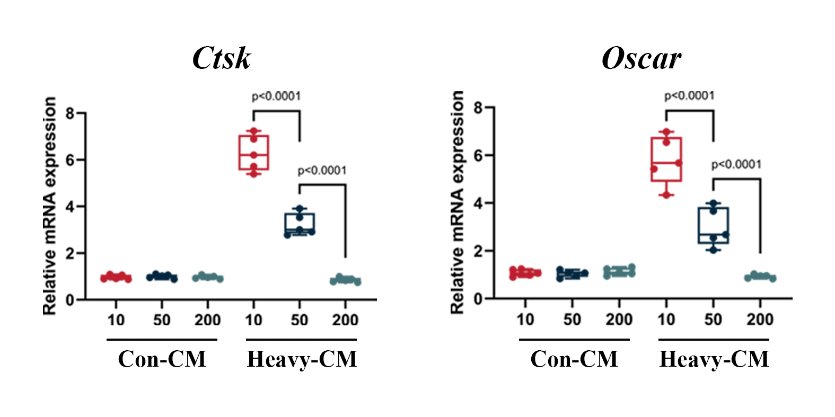


**Appendix Figure 7.** The Quantitative RT-PCR analysis was performed to examine the mRNA levels of *Ctsk* and *Oscar*. Statistical comparison was performed using one-way ANOVA with Tukey’s post hoc test. p < 0.05 was considered statistically significant. n = 5 for each group. All data were presented as mean ± SEM.


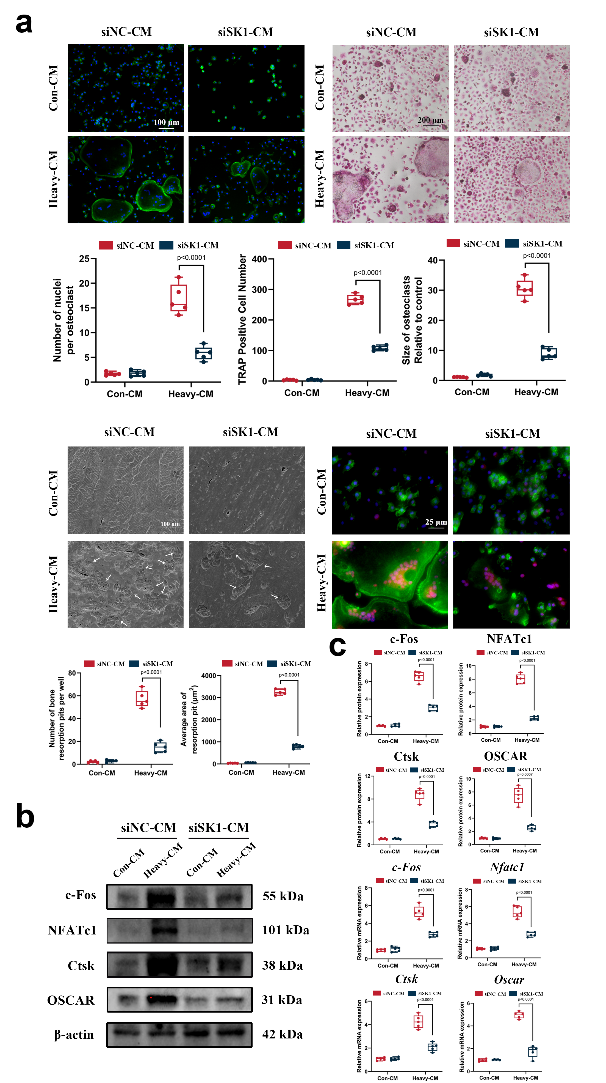


**Appendix Figure 8.** Knockdown of SphK1 in IDG-CM6 cells produced effects on osteoclastogenesis. **a** Representative images of TRAP staining. The arrows indicated osteoclasts. Scale bar = 200 μm. Representative images of F-actin ring staining. The arrows indicated osteoclasts. Scale bar = 100 μm. Representative immunofluorescence images of NFATc1 level in osteoclasts. Cytoskeleton, green; NFATc1, red; Nuclei, blue. Scale bar = 25 μm. The SEM of resorption pits on bovine slices seeded with differentiating osteoclasts were also performed, the white arrows indicated the resorption lacunae on bovine slices. Scale bar = 100 μm. Quantitative analysis of number of nuclei per osteoclast and quantification of number and relative size of TRAP positive multinucleated osteoclasts per well, as well as the quantification of number and average area of resorption pits, were performed and values were normalized to those in the Con-CM with si-NC group. **b, c** The protein expression of c-Fos, NFATc1, Ctsk and OSCAR in BMMs were examined by western blot analysis using GAPDH as a loading control. The quantitative analysis of protein expression of c-Fos, NFATc1, Ctsk and OSCAR was also performed. Quantitative RT-PCR analysis was performed to examine the mRNA levels of *c-Fos*, *Nfatc1*, *Ctsk* and *Oscar*. Statistical comparison was performed using two-tailed Student’s t test with Tukey’s post hoc test. p < 0.05 was considered statistically significant. n = 5 for each group. All data were presented as mean ± SEM.


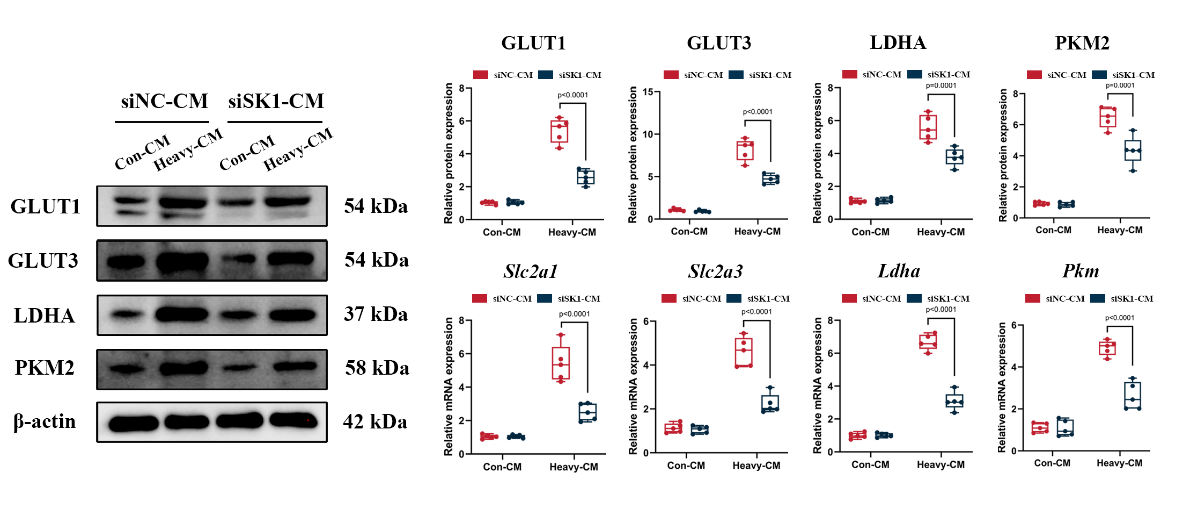


**Appendix Figure 9.** The protein expression of GLUT1, GLUT3, LDHA and PKM2 in BMMs were examined by western blot analysis using β-actin as a loading control. The quantitative analysis of protein expression of GLUT1, GLUT3, LDHA and PKM2 was also performed. Quantitative RT-PCR analysis was performed to examine the mRNA levels of *Slc2a1*, *Slc2a3*, *Ldha* and *Pkm*. Statistical comparison was performed using two-tailed Student’s t test with Tukey’s post hoc test. p < 0.05 was considered statistically significant. n = 5 for each group. All data were presented as mean ± SEM.


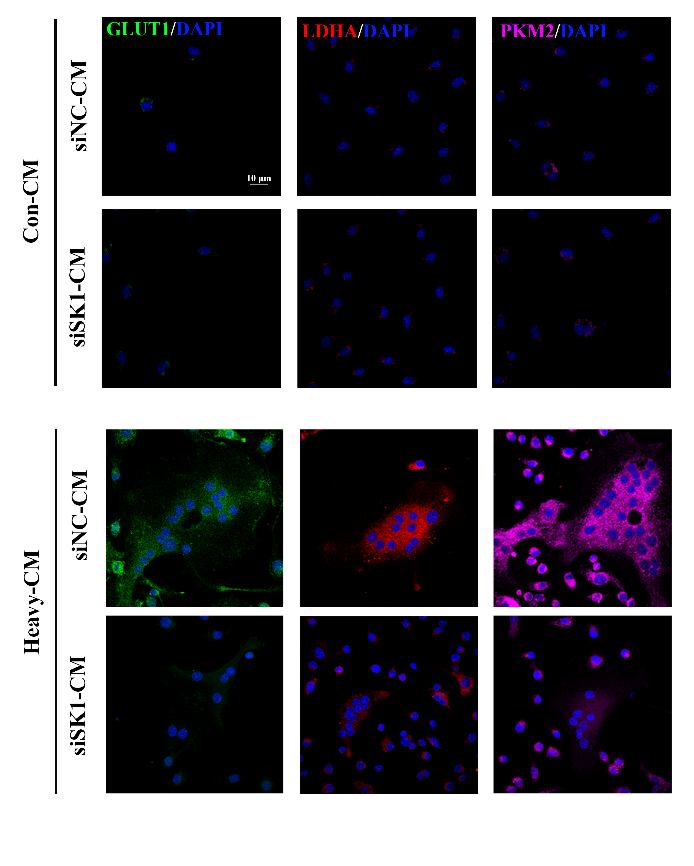


**Appendix Figure 10.** Representative immunofluorescence images of GLUT1, LDHA and PKM2 levels in osteoclasts. GLUT1, green; LDHA, red; PKM2, magenta; Nuclei, blue. Scale bar = 10 μm.


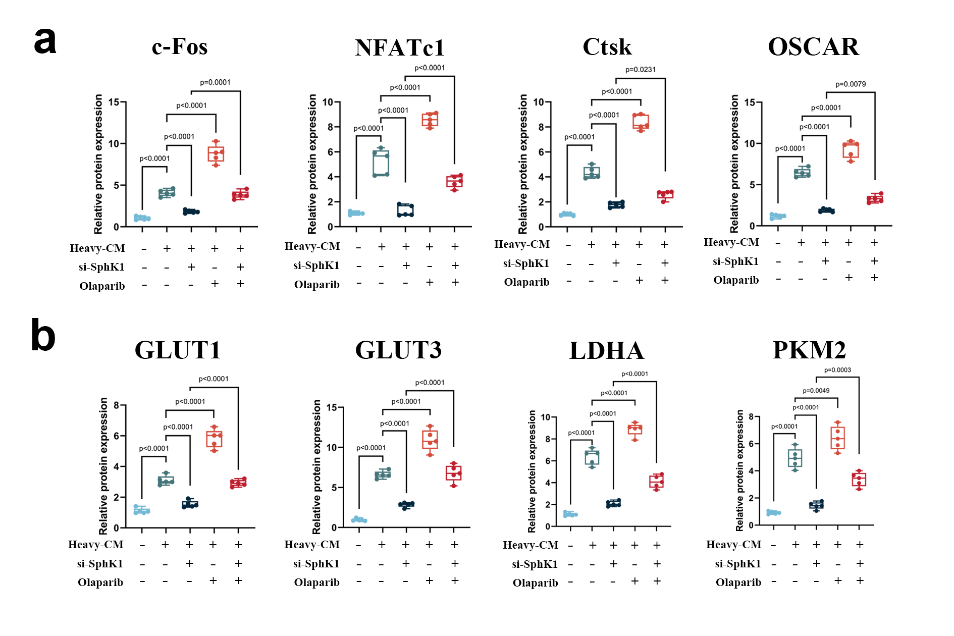


**Appendix Figure 11.** **a** The quantitative analysis of protein expression of c-Fos, NFATc1, Ctsk and OSCAR was also performed. **b** The quantitative analysis of protein expression of GLUT1, GLUT3, LDHA and PKM2 was also performed. Statistical comparison was performed using one-way ANOVA with Tukey’s post hoc test. p < 0.05 was considered statistically significant. n = 5 for each group. All data were presented as mean ± SEM.


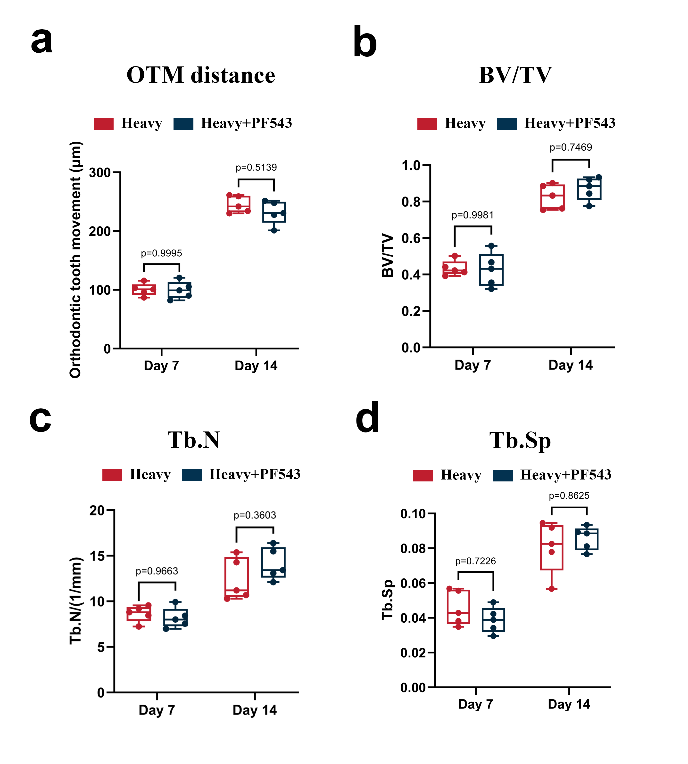


**Appendix Figure 12.** Inhibition of SphK1 did not interrupt OTM rate and bone remodeling. **a** The distance of OTM at day 14 was measured. **b-d** Parameters including bone volume/total volume (BV/TV), trabecular number (Tb.N) and trabecular spacing (Tb.Sp) were examined at both day 7 and day 14. Statistical comparison was performed using two-tailed Student’s t test with Tukey’s post hoc test. p < 0.05 was considered statistically significant. n = 5 for each group. All data were presented as mean ± SEM.


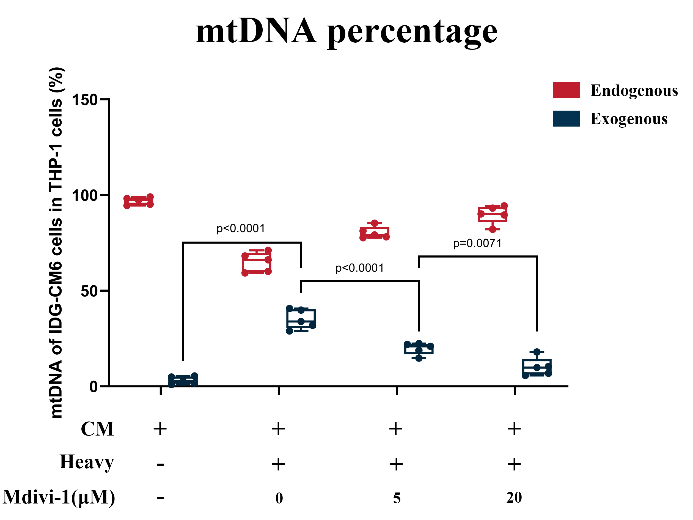


**Appendix Figure 13.** Genetic evidence for force- and mitophagy-dependent mitochondrial transfer from mouse IDG-CM6 cells to human THP-1 cells. Data are presented as the percentage of mouse mtDNA relative to the total (mouse + human) mtDNA signal. Statistical comparison was performed using two-tailed Student’s t test with Tukey’s post hoc test. p < 0.05 was considered statistically significant. n = 5 for each group. All data were presented as mean ± SEM.


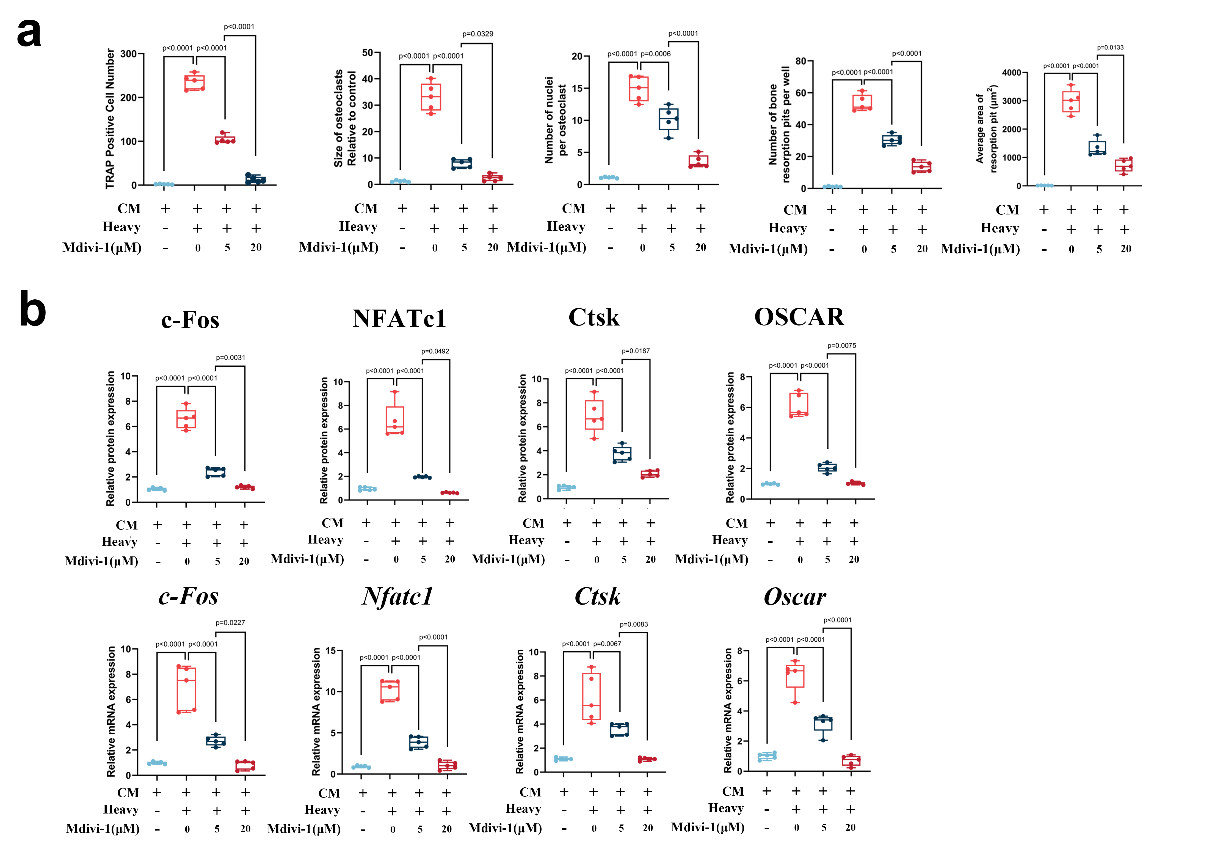


**Appendix Figure 14.** **a** Quantitative analysis of number of nuclei per osteoclast and quantification of number and relative size of TRAP positive multinucleated osteoclasts per well, as well as the quantification of number and average area of resorption pits, were performed and values were normalized to those in the CM-only group. **b** The quantitative analysis of protein expression of c-Fos, NFATc1, Ctsk and OSCAR was also performed. Quantitative RT-PCR analysis was performed to examine the mRNA levels of *c-Fos*, *Nfatc1*, *Ctsk* and *Oscar*. Statistical comparison was performed using one-way ANOVA with Tukey’s post hoc test. p < 0.05 was considered statistically significant. n = 5 for each group. All data were presented as mean ± SEM.

**Appendix Table**

**Appendix Table 1. Sequences of primers used in qRT-PCR**

| Gene | Forward primer sequence (5′–3′) | | Reverse primer sequence (5′–3′) | |  |
| --- | --- | --- | --- | --- | --- |
| *c-Fos* | | 5’ TCTCTAGTGCCAACTTTATCCC 3’ | | 5’ GAGATAGCTGCTCTACTTTGCC 3’ | |
| *Nfatc1* | | 5’ GAGAATCGAGATCACCTCCTAC 3’ | | 5’ TTGCAGCTAGGAAGTACGTCTT 3’ | |
| *Ctsk* | | 5’ GCTTGGCATCTTTCCAGTTTTA 3’ | | 5’ CAACACTGCATGGTTCACATTA 3’ | |
| *Oscar* | | 5’ GGAATGGTCCTCATCTCCTT 3’ | | 5’ TCCAGGCAGTCTCTTCAGTTT 3’ | |
| *Slc2a1* | | 5’ CACTGTGGTGTCGCTGTTTG 3’ | | 5’ AAAGATGGCCACGATGCTCA 3’ | |
| *Slc2a3* | | 5’ GTGACTGTGCTGGAGCTCTT 3’ | | 5’ ATCCCAGAGAGCTGCTGAGA 3’ | |
| *Ldha* | | 5’ AGTAAGTCCTCAGGCGGCTA 3’ | | 5’ TGAGGGTTGCCATCTTGGAC 3’ | |
| *Pkm* | | 5’ TCACCCTGGACAACGCTTAC 3’ | | 5’ AGTCAGCGCCTTTCTCCTTC 3’ | |
| *Pink1* | | 5’ TATCTCGGCAGGTTCCTCCA 3’ | | 5’ AAGCTGCTTGGGACCATCTC 3’ | |
| *Prkn* | | 5’ CCTGCAAACAAGCAACCCTC 3’ | | 5’ TCACCACTCATCCGGTTTGG 3’ | |
| *Dnm1l* | | 5’ GGCAACTGGAGAGGAATGCT 3’ | | 5’ CTTGCAACTGGAACTGGCAC 3’ | |
| *Fis1* | | 5’ CAGAGACGAAGCTGCAAGGA 3’ | | 5’ CTTTGCTCCCTTTGGGCAAC 3’ | |
| *Atg5* | | 5’ AAGCAGCTCTGGATGGGACTG 3’ | | 5’ CCGCTCCGTCGTGGTCTG 3’ | |
| *Atg7* | | 5’ ACCATGCAGGGAGCTAGAGA 3’ | | 5’ CCCATGCCTCCTTTCTGGTT 3’ | |
| *Becn1* | | 5’ GGACGTGGAGAAAGGCAAGA 3’ | | 5’ GAACTGTGAGGACACCCAGG 3’ | |
| *Gapdh* | | 5’ AGGTGAAGGTCGGAGTCAAC 3’ | | 5’ CGCTCCTGGAAGATGGTGAT 3’ | |
| *Actb* | | 5’ AGCCATGTACGTAGCCATCC 3’ | | 5’ GACTCCATCACAATGCCAGT 3’ | |
